# The effect of mutation on an aggregation-prone protein: An in vivo, in vitro, and in silico analysis

**DOI:** 10.1073/pnas.2200468119

**Published:** 2022-05-25

**Authors:** N. Guthertz, R. van der Kant, R. M. Martinez, Y. Xu, C. Trinh, B. I. Iorga, F. Rousseau, J. Schymkowitz, D. J. Brockwell, S. E. Radford

**Affiliations:** ^a^Astbury Centre for Structural Molecular Biology, School of Molecular & Cellular Biology, Faculty of Biological Sciences, University of Leeds, Leeds LS2 9JT, United Kingdom;; ^b^Switch Laboratory, VIB-KU Leuven Center for Brain & Disease Research, 3000 Leuven, Belgium;; ^c^Department of Cellular and Molecular Medicine, KU Leuven, 3000 Leuven, Belgium;; ^d^Université Paris-Saclay, CNRS UPR 2301, Institut de Chimie des Substances Naturelles, 91198 Gif-sur-Yvette, France

**Keywords:** amyloid, aggregation, evolution

## Abstract

Aggregation of initially stably structured proteins is involved in more than 20 human amyloid diseases. Despite intense research, however, how this class of proteins assembles into amyloid fibrils remains poorly understood, principally because of the complex effects of amino acid substitutions on protein stability, solubility, and aggregation propensity. We address this question using β_2_-microglobulin (β_2_m) as a model system, focusing on D76N-β_2_m that is involved in hereditary amyloidosis. This amino acid substitution causes the aggregation-resilient wild-type protein to become highly aggregation prone in vitro, although the mechanism by which this occurs remained elusive. Here, we identify the residues key to protecting β_2_m from aggregation by coupling aggregation with antibiotic resistance in *E. coli* using a tripartite β-lactamase assay (TPBLA). By performing saturation mutagenesis at three different sites (D53X-, D76X-, and D98X-β_2_m) we show that residue 76 has a unique ability to drive β_2_m aggregation in vivo and in vitro. Using a randomly mutated D76N-β_2_m variant library, we show that all of the mutations found to improve protein behavior involve residues in a single aggregation-prone region (APR) (residues 60 to 66). Surprisingly, no correlation was found between protein stability and protein aggregation rate or yield, with several mutations in the APR decreasing aggregation without affecting stability. Together, the results demonstrate the power of the TPBLA to develop proteins that are resilient to aggregation and suggest a model for D76N-β_2_m aggregation involving the formation of long-range couplings between the APR and Asn76 in a nonnative state.

## Significance

Protein aggregation is a major problem for human health. However, our understanding of how folded proteins aggregate into amyloid lags behind. Using the tripartite β-lactamase assay (TPBLA) with our test protein, β_2_-microglobulin (β_2_m), we show the ability to differentiate the behavior of single-point variants and highlight the remarkable sensitivity to the identity of the residue at position 76. After evolving the aggregation-prone protein, D76N-β_2_m, the only mutations able to improve D76N-β_2_m behavior in vivo involve residues in a single 7-residue sequence of the protein. Further characterization in vitro shows that a single-point mutant in this region can abolish D76N-β_2_m aggregation.

Protein misfolding and aggregation are involved in more than 50 human diseases, some of which are the most debilitating disorders that threaten human health today (1). Despite intense research, understanding why some proteins aggregate while others are resilient and predicting how alterations in the sequence affect aggregation and pathogenicity remain a challenge (2, 3). Proteins that aggregate into amyloid fibrils associated with disease include intrinsically disordered proteins (IDPs) and peptides, such as α-synuclein and amyloid beta (Aβ), as well as folded proteins such as transthyretin (TTR), antibody light chains, and β_2_-microglobulin (β_2_m) (4). In each case, aggregation results in the formation of fibrils with a cross-β structure that is canonical of amyloid fibrils (4). For both types of protein precursor, single amino acid changes via mutation or posttranslational modification can have dramatic effects on aggregation propensity and can result in familial or early-onset amyloid disease. For example, the single substitution S20G in the human islet amyloid polypeptide (IAPP) is associated with early onset of type II diabetes (5), while an array of single amino acid changes in Aβ are associated with familial Alzheimer’s disease (6, 7). More extreme examples include the 120 mutations in TTR that have been implicated in autosomal-dominant amyloid disease (8) and the large array of variants associated with light chain amyloidosis innate to each germline. These arise as a consequence of somatic hypermutation (9, 10). Another example is β_2_m, in which aggregation of the wild-type (WT) protein is associated with the deposition of amyloid fibrils in the joints of patients undergoing long-term renal dialysis (dialysis-related amyloidosis [DRA]) (11–13). More recently, two single-point variants of this protein, D76N and V27M, have been identified in amyloid deposits in the viscera or tongue, respectively, with the former occurring in the absence of kidney disease (14, 15).

Several algorithms have been developed that aim to predict the outcome of protein sequence changes on aggregation propensity (reviewed in ref. 3). Each focuses on a specific set or sets of parameters, such as protein solubility (16), stability (if initially folded) (17), propensity to form β-aggregates or β-zippers (18), frustration (19, 20), or a combination of these factors (21–24). Predicting the presence of aggregation-prone regions (APRs) within a protein sequence (a contiguous sequence typically between 5 and 15 residues long) can be achieved with confidence using these algorithms (25, 26). However, when an APR is contained within a long IDP or is embedded within a globular protein, it remains challenging to predict whether a particular protein sequence will assemble into amyloid, since aggregation depends on each of these criteria (and others) (3).

**Fig. 1.**
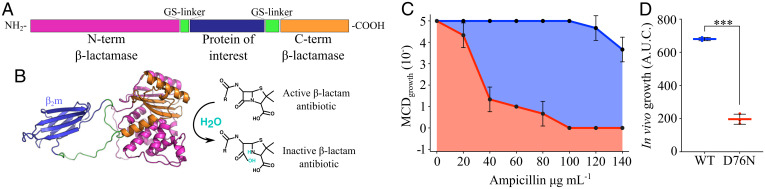
The TPBLA. (*A*) The test protein (blue) is inserted into a 29-residue glycine/serine-rich linker (green, 11 amino acids at the N-terminal and 18 amino acids at the C-terminal of the test protein) separating the two domains of the *E. coli* enzyme TEM-1 β-lac (pink/orange). (*B*) Structural model of the fusion protein expressed in the TPBLA (*Left*). If the two domains of β-lac are able to dock together, the enzyme will be active and can hydrolyze β-lactam antibiotics (*Right*). (*C*) Antibiotic survival curves showing the maximal cell dilution (MCD_growth_) allowing growth on solid medium over a range of ampicillin concentrations. The AUCs for a resistant (blue) and more sensitive strain (red) expressing WT- or D76N-β_2_m, respectively, are shown. An MCD_growth_ of 10^-1^ indicates no growth of an undiluted sample. (*D*) The antibiotic survival AUC yields a single value that can be used to compare the effect of sequence changes in the guest protein on the bacterial survival curves. Asterisks denote significance: ***P = 0.002 (t-test: paired two sample for means, two-tail). The error bars in *C* and *D* represent one SD (*n* = 3 biologically independent experiments), where each point corresponds to one experiment.

Recent developments in experimental methods using evolution and selection approaches have the potential to provide large datasets of protein sequences with different behavior in vitro or in a cellular setting (3). We have previously reported one such approach, the tripartite β-lactamase assay (TPBLA) (27, 28)—which has been used to enhance the thermodynamic stability of globular proteins (29, 30)—to rank the aggregation propensity of variants of Aβ and IAPP (28), to screen for small-molecule inhibitors of aggregation (28), and to evolve aggregation-resistant proteins of relevance to biopharma (27). In the TPBLA, a peptide or protein of interest is fused in-frame between the two domains of TEM-1 (Temoneira-1) β-lactamase (β-lac), and the fusion protein is expressed in the periplasm of *E. coli* (Fig. 1*A*). Antibiotic resistance of the resulting bacteria (Fig. 1*B*), measured by an in vivo growth score in the presence of different concentrations of ampicillin, is then used to reflect the inherent characteristic (stability/solubility/aggregation propensity or other) of the inserted sequence since a paucity in any such characteristic will reduce the concentration of folded β-lac in the periplasm and, hence, the ability of the bacteria to grow in the presence of an antibiotic. The TPBLA is tolerant of the insertion of peptides, as well as longer IDPs and structured protein domains (single- and two-domain proteins up to 40 kDa in size have been successfully analyzed to date) (27, 28, 30). It also has the advantage that the periplasm is oxidizing, enabling analysis of proteins and peptides that contain disulphide bonds. In the case of short peptides and IDPs, the TPBLA yields a relatively simple readout since the overriding feature dominating selection is the inherent aggregation propensity of the sequence (28). In the case of stably folded proteins, the TPBLA provides a readout that correlates with protein solubility and stability (27). For proteins that are folded but aggregation prone, such as the folded precursors of amyloid disease, the readout is potentially complex, as many parameters can influence the in vivo growth score, including *inter alia* protein stability, aggregation propensity, solubility, and other characteristics.

**Fig. 2.**
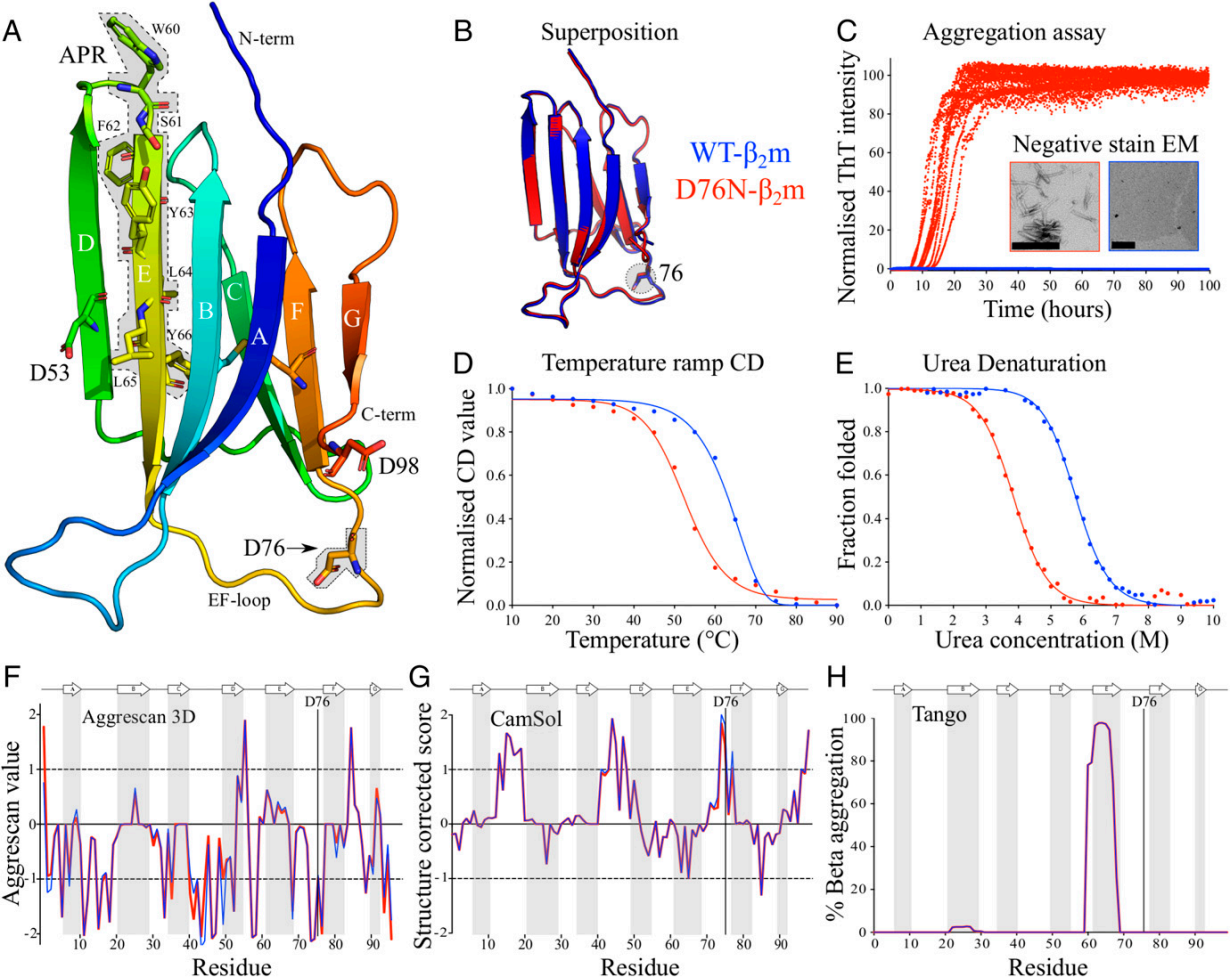
Comparison of the structure, stability, and amyloid propensity of WT- and D76N-β_2_m. (*A*) Crystal structure of WT-β_2_m (PDB [Protein Data Bank] 1LDS; ref. 61) highlighting Asp53, Asp76, Asp98, and the APR (residues 60 to 66 with gray background). The single disulphide bond linking Cys25 to Cys80 is also shown. Residue 76 is highlighted in stick and with a gray box. (*B*) Superposition of the crystal structure of WT- (PDB 1LDS; ref. 61) and D76N-β_2_m (PDB 4FXL; ref. 15) (RMSD of 0.33 Å over all heavy atoms). (*C*) ThT kinetics of the aggregation of WT- (blue) and D76N-β_2_m (red) (40 µM protein, 25 mM sodium phosphate pH 6.2, 115 mM NaCl, and 37 °C with agitation). Note the same color coding is used in all figure parts (WT- [blue] and D76N-β_2_m [red]). Ten replicates are shown. (*Inset*) Negative-stain EM micrographs of the endpoint of each sample are shown. Scale bar: 200 nm. (*D*) Apparent stability of WT- and D76N-β_2_m (20 µM) measured using thermal denaturation by far UV CD (25 mM sodium phosphate pH 6.2). (*E*) The thermodynamic stability monitored by urea titration using tryptophan fluorescence (see *SI Appendix*, Table S1). (*F*–*H*) Predictions of the aggregation propensity of WT- and D76N-β_2_m using (*F*) structurally corrected Aggrescan 3D 2.0 (23, 24) (values > +1 indicate APRs and < −1 indicate aggregation-resistant regions [dotted black lines]), (*G*) structure-corrected CamSol (16) (values > +1 indicate soluble and < −1 indicate insoluble regions [dotted black lines]), and (*H*) sequence-based Tango (18) (values >5% correspond to an aggregation-prone sequence). Light gray vertical bars highlight the β-strands in the native structure. The PDB codes used are 1LDS (61) for WT-β_2_m and 4FXL (15) for D76N-β_2_m, where residue M0 was removed and residues R97, D98, and M99 were added to 1LDS (61) to ensure a similar number of residues to compare with D76N-β_2_m.

Here, we report the use of the TPBLA to explore the role of the protein sequence in determining the aggregation of the naturally amyloidogenic protein, β_2_m. β_2_m is 99 residues in length (10.8 kDa) and has a seven-stranded β-sandwich immunoglobulin fold that is stabilized by a single disulphide bond linking Cys25 and Cys80 (31, 32) (Fig. 2*A*). The protein forms the noncovalently bound light chain of the major histocompatibility complex class 1 (MHC 1) (33) and is important for antigen presentation (34, 35). In healthy individuals, β_2_m dissociates from the MHC 1 as part of its catabolic cycle, whereupon it is cleared from the serum via the kidneys (11). However, in individuals with renal failure, β_2_m is no longer efficiently removed from the blood, resulting in an increased serum β_2_m concentration and the formation of amyloid fibrils that deposit in the joints in the disorder known as DRA (11–13).

In 2012, the first familial β_2_m variant involved in hereditary amyloid disease was identified (15). The amyloid deposits were found in the visceral organs and contained the variant D76N-β_2_m, despite the individuals being heterozygous for the mutation and having normal serum β_2_m levels (15). Subsequent analysis revealed that D76N-β_2_m is less stable than WT-β_2_m, but why the substitution of Asp to Asn in a solvent-exposed loop enhances the amyloid propensity of the protein remains mysterious since other amino acid substitutions that reduce stability to a similar, or an even greater, extent (e.g., V37A-β_2_m) have no effect on protein aggregation (36). Substitution of each of the other six Asn residues in the β_2_m sequence to Asp does not induce amyloid formation, highlighting a unique role of residue 76 in controlling aggregation of the protein (37). While aggregation of WT-β_2_m is driven by the formation of a partially folded state containing a nonnative *trans* Pro32 (known as the I_T_-state) (38), the population of the I_T_-state is not enhanced for the more rapidly aggregating D76N-β_2_m, suggesting that WT- and D76N-β_2_m aggregate through distinct mechanisms (31). Indeed, it has been hypothesized that a different species, possibly a native-like state (named the N*-state), could be responsible for D76N-β_2_m aggregation (39), highlighting the complex relationship of protein sequence and aggregation mechanism for this protein.

To explore the relationship between sequence and amyloid propensity for D76N-β_2_m in more detail, we here combined in vivo, in vitro, and in silico methods to search for amino acid substitutions that reduce the aggregation propensity of the protein. Using saturation mutagenesis of residue 76 analyzed by the TPBLA alongside analysis of saturation libraries of two other solvent-exposed Asp residues (D53 and D98) and in vitro determination of the structural properties, stability, and aggregation rate of the D76X variants, we show a remarkable specificity of the identity of residue 76 in determining β_2_m aggregation in vitro and in vivo that could not be recapitulated by in silico prediction methods. Random mutagenesis and selection for enhanced antibiotic resistance using the TPBLA was then used to screen for amino acid substitutions able to reduce D76N-β_2_m aggregation. The results revealed a single region of the β_2_m sequence, involving residues 60 to 66, that is required for D76N-β_2_m aggregation. This region has a high predicted and experimentally validated aggregation propensity and has been shown previously to control the rate of aggregation of acid unfolded WT-β_2_m into amyloid by tailoring its conformational dynamics (40). The results are consistent with a model for D76N-β_2_m aggregation in which the substitution of Asp76 with Asn destabilizes the native protein and increases the population of nonnative conformers that have an enhanced aggregation propensity, in contrast with WT-β_2_m and its truncation variant ΔN6 that aggregate via their I_T_-states (41). The results highlight the power of protein evolution and selection methods to generate proteins able to resist aggregation that cannot be predicted using current algorithms. Such data could be used in the future to inform in silico methods better able to predict aggregation of amyloidogenic proteins.

## Results

### Experimental and Predicted Differences in Stability and Aggregation of WT- and D76N-β_2_m.

As previously described (15), WT- and D76N-β_2_m have similar structures (root-mean-square deviation [RMSD] of 0.33 Å over all heavy atoms) (Fig. 2*B*) but profoundly different abilities to aggregate into amyloid fibrils in vitro (the half time of aggregation [T_half_] for D76N-β_2_m is 13.7 ± 1.6 h, while WT-β_2_m does not aggregate over the course of this experiment) (Fig. 2*C*). Substitution of Asp76 with Asn reduces protein stability significantly (apparent melting temperature [T_m;app_] of 65.2 ± 0.4 °C and 53.8 ± 0.2 °C, and ΔG°_UN_ -29.2 ± 0.2 kJ mol^−1^ and -18.6 ± 0.2 kJ mol^−1^, for WT- and D76N-β_2_m, respectively) (Fig. 2 *D* and *E* and *SI Appendix*, Table S1), consistent with previous reports (15, 37, 39). FoldX (17) predicts that substitution of Asp to Asn at position 76 should destabilize β_2_m by 0.15 kJ mol^−1^, a significantly smaller value than that observed experimentally (ΔΔG°_UN_ [D76N – WT-β_2_m] = 9.5 kJ mol^−1^). These dramatic differences in stability and aggregation propensity are surprising and difficult to predict or rationalize, as residue 76 is solvent exposed and found in the loop linking β-strands E and F (the EF loop) in the native structure of β_2_m (Fig. 2*A*). Notably, other β_2_m variants with similar stability to D76N-β_2_m, or in which stability is reduced even further, such as murine-β_2_m (42) or V37A-β_2_m (36), do not aggregate in vitro under the conditions used, ruling out a simple correlation between protein stability and aggregation, as has been observed for other proteins with an immunoglobulin fold, such as variants of antibody light chains (30, 43). To explore the origins of the observed marked difference in aggregation of WT- and D76N-β_2_m, online prediction algorithms were also used (Fig. 2 *F*–*H*). The prediction algorithms—Aggrescan 3D 2.0 (23, 24) (predicts protein aggregation based on protein sequence, stability, and structure), CamSol (16, 44) (predicts protein solubility based on sequence and structure), and Tango (18) (predicts aggregation based on sequence alone)—each failed to show a difference in predicted behavior of the two proteins. Further online predictors/algorithms were also used without success (*SI Appendix*, Table S2).

### Saturation Mutagenesis at Positions 53, 76, and 98 Measured Using the TPBLA.

To confirm the ability of the TPBLA to detect changes in β_2_m aggregation, the assay was performed on WT- and D76N-β_2_m by inserting these sequences individually into the linker of β-lac. Each variant exhibited dramatically different scores in the TPBLA (Fig. 1*C*), with D76N-β_2_m giving a significantly lower score compared to WT-β_2_m (enumerated as the area under the MCD_growth_ curve [AUC] of 680 ± 10 AUC and 191 ± 31 AUC for WT- and D76N-β_2_m, respectively), consistent with previous results (28) (Fig. 1*D*). These data highlight the sensitivity of the TPBLA to detect the effect of single-point amino acid substitutions on β_2_m behavior.

**Fig. 3.**
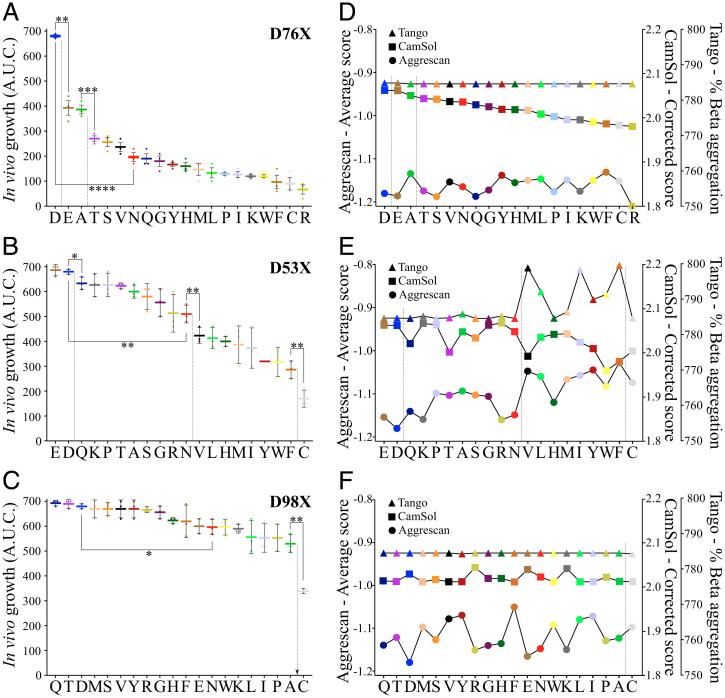
Saturation mutagenesis of β_2_m at positions 76, 53, or 98. (*A*–*C*) In vivo growth score (AUC) of the 20 (*A*) D76X-, (*B*) D53X-, or (*C*) D98X-β_2_m variants. In each case, the residues are ordered from highest to lowest in vivo growth score. Data represent mean values (*n* = 3 biologically independent experiments), where each point corresponds to one experiment. The error bar shows one SD from the mean. Asterisks denote significance (t-test: paired two sample for means, two-tail) where *P = 0.03, **P = 0.01, ***P = 0.003, and ****P = 0.002. (*D*–*F*) Aggrescan 3D 2.0 (23, 24), structurally corrected CamSol (16), and sequence-based Tango (18) score for each variant. The vertical black lines demark the different groups that are statistically different (*P* > 0.05). Each amino acid type is colored the same in the six plots and ordered in the same way for D76X- (*A* and *D*), D53-X (*B* and *E*), or D98X- (*C* and *F*). Note that the Asp (“D”) corresponds to the amino acid in WT-β_2_m.

Next, each of the 20 natural amino acids was placed at position 76 in the protein sequence using mutagenesis, and the in vivo growth score of bacteria expressing each of these variants was measured over a range of ampicillin concentrations (0 to 140 µg ml^−1^) (*SI Appendix*, Fig. S1*A***)**. The results (Fig. 3*A*) were striking, showing that D76-β_2_m (corresponding to WT-β_2_m) has by far the highest score (∼ 700 AUC), consistent with this protein being highly resilient to aggregation and/or degradation in the *E. coli* periplasm. A second group of residues, with a significantly reduced score compared to Asp76, includes the D76E- and D76A-β_2_m variants (∼ 400 AUC [*P* = 0.01 compared with WT-β_2_m]), while a third group contains the remaining 17 D76X-β_2_m variants, which have significantly decreased scores (*P* = 0.003 compared with D76E/A-β_2_m) (Fig. 3*A*). This last group of variants has gradually decreasing scores (ranging from ∼ 300 [for D76T-β_2_m] to ∼ 50 AUC [for D76R-β_2_m]). Notably, the substitution D76N lies in the middle of this third group, but its score is not significantly different from those of other variants in this group. Overall, the results show that the presence of a negatively charged Asp protects β_2_m from aggregation, at least as judged by the TPBLA, with the negatively charged D76E-β_2_m or neutral D76A-β_2_m providing some, albeit more limited, protection as judged by this assay.

To better understand the importance of substitutions at residue 76 on the behavior of β_2_m, two additional Asps were selected for analysis by the TPBLA: Asp53 (a solvent-exposed residue in the center of the edge D-strand of the β-sandwich fold) and Asp98 (also solvent exposed and the penultimate residue of the protein) (Fig. 2*A*). Comparison of the MCD_growth_ curves of D76N-, D53N-, and D98N-β_2_m showed that each has a significantly different score compared to WT-β_2_m (*SI Appendix*, Fig. S1 *A*–*C*), with D98N-β_2_m having the highest score (*∼* 600 AUC), followed by D53N-β_2_m (*∼* 500 AUC), and D76N-β_2_m with the lowest score (*∼* 200 AUC) (*P* < 0.04 for the three variants compared with WT-β_2_m; *SI Appendix*, Fig. S2*A*). This indicates that an Asp-to-Asn substitution at these sites affects β_2_m differently, depending on the residue’s position in the native structure. Since these variants all involve an Asp-to-Asn substitution at a solvent-exposed site, the difference in MCD_growth_ of these variants cannot be attributed to differences in overall protein charge or solubility. Accordingly, no significant difference in protein solubility or aggregation propensity was predicted for these three variants using the algorithms Tango (18), CamSol (16), or Aggrescan 3D (23, 24) (*SI Appendix*, Figs. S2*B* and S3 *D*–*F*).

Saturation mutagenesis was next carried out at positions 53 and 98, and the MCD_growth_ of the resulting 40 variants was determined using the TPBLA (*SI Appendix*, Fig. S1 *B* and *C*). By contrast with amino acid substitutions at position 76, which result in proteins that are sensitive to the identity of the amino acid at that site, residue 98 is resilient to substitution, with little change in the in vivo growth score for 19 of the 20 sequences (the sole exception is D98C, which could be explained by the formation of an incorrect intra- or intermolecular disulfide bond) (*SI Appendix*, Fig. S1*C*). Notably, Cys substitution at residues 53 and 76 also results in a reduced in vivo growth score (Fig. 3 *A* and *B*). Interestingly, the D53X-β_2_m series resulted in proteins with a behavior in the TPBLA distinct to those of the resilient D98X-β_2_m or sensitive D76X-β_2_m series, with a gradual change in in vivo growth score across all 20 residue types (*SI Appendix*, Fig. S1*B*). Consistent with this, a poor correlation is observed between the in vivo growth scores of D98X-β_2_m and D53X-/D76X-β_2_m, as judged by their rank-based Spearman correlations of 28 and 43%, respectively (*SI Appendix*, Fig. S3).

Whether a protein aggregates depends on the balance of many factors—including protein structure, stability, solubility, and inherent aggregation propensity—and can be modulated by the sequence (such as the inclusion of charges or gatekeeper residues local to an APR; ref. 45). The predicted properties of the 20 sequence variants at positions 76, 53, and 98 were determined using the algorithms Tango (18), CamSol (16), and Aggrescan 3D (23, 24) and compared with the in vivo growth scores of the variants at each position. For D76X-β_2_m, a perfect correlation (*r* = 100% using a rank-based Spearman correlation; *SI Appendix*, Fig. S4) was observed between protein solubility and in vivo growth score, while Tango (18) and Aggrescan3D (23, 24) did not correlate (Fig. 3*D*). For D98X-β_2_m, the in vivo growth score and the predictions of all three algorithms were not significantly affected by substitution, suggesting that the identity of this residue plays little or no role in determining aggregation in vivo or in silico (Fig. 3*F*). For D53X-β_2_m, where the substitution site is at an edge-strand, a different scenario ensues. For this series of variants, protein behavior can be divided roughly into four groups, depending on amino acid type (with a statistically significant difference between each group: *P* < 0.05; Fig. 3*B*). The first group contains WT- and D53E-β_2_m, indicating that increasing the size of the acidic sidechain does not affect the protein’s behavior. The second group contains mostly positively charged or polar residues (D53Q-, D53K-, D53T-, D53S-, D53R-, and D53N-β_2_m), as well as D53A-, D53P-, and D53G-β_2_m. The third group contains the remaining hydrophobic residues (D53V-, D53L-, D53H-, D53M-, D53I-, D53Y-, D53W-, and D53F-β_2_m), while the fourth group contains only D53C-β_2_m. Aggrescan3D (23, 24) and CamSol (16) were able to capture the rank order and in vivo growth scores of the D53X-β_2_m variants, with Tango performing less well (Fig. 3*E* and *SI Appendix*, Fig. S5). Interestingly, an inverse correlation was observed between β-strand propensity and the rank order of the in vivo growth score (*r* = 75% using a rank-based Spearman correlation) for the variants at residue 53 (i.e., residues with a higher β-strand propensity resulted in a lower in vivo growth score; *SI Appendix*, Fig. S5 *E* and *F*). This observation could be rationalized by the known selection against structures with edge β-strands that are solvent exposed and straight, presumably as a protective mechanism against in vivo aggregation (*SI Appendix*, Fig. S5*I*) (46). Overall, the results portray the sensitivity of the TPBLA to single amino acid substitutions in a protein sequence and reveal the different responses to substitutions at the three different sites in β_2_m analyzed here.

### Stability and Aggregation of Purified Proteins Mutated at Position 76.

**Fig. 4.**
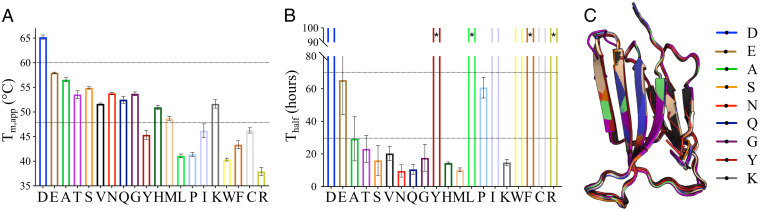
Stability, aggregation, and crystal structures of the D76X-β_2_m variants. (*A*) Protein stability represented by T_m;app_ was determined for the 20 D76X-β_2_m variants using temperature ramp monitored by far-UV CD at 216 nm (*SI Appendix*, *Materials and Methods*) The error bars represent the fitting error. The dashed line at 60 °C identifies proteins (WT-β_2_m) whose high T_m;app_ prevents aggregation, while the line at 48 °C indicates the T_m;app_ below which the D76X-β_2_m variants fail to aggregate over the course of these experiments or do slow very slowly (note: D76M-β_2_m has a thermal stability slightly above 48 °C). (*B*) Protein aggregation rate represented by T_half_ calculated for all D76X-β_2_m variants using ThT fluorescence measurements. The error bars represent one SD of the mean from 8 to 10 technical repeats (two biological replicates were performed, but only one is shown here). The dotted lines separate the stable versus unstable variants in *A* and the aggregation-prone and aggregation-resilient variants in *B*. Each amino acid type is colored the same in *A* and *B*, and the variants are ordered identically to Fig. 3*A* (i.e., high to low in vivo growth score). “D” corresponds to WT-β_2_m. The asterisk in *B* corresponds to variants that started to aggregate just before the endpoint (100 h). (*C*) Superposition of the crystal structures of nine β_2_m variants: WT- (PDB 1LDS; ref. 61), D76A- (PDB 7NMO), D76E- (PDB 7NMC), D76S- (PDB 7NMR), D76N- (PDB 4FXL; ref. 15), D76Q- (PDB 7NMV), D76G- (PDB 7NMT), D76K- (PDB 7NN5), and D76Y-β_2_m (PDB 7NMY) showing that the amino acid substitutions at residue 76 do not alter the structure of the proteins significantly, including the EF loop that contains residue 76.

To better understand the effect of the amino acid substitutions at residue 76 on structure, stability, and aggregation potential, the 20 D76X-β_2_m variants were cloned (in the absence of β-lac), expressed in *E. coli*, and purified (*SI Appendix*, *Materials and Methods*). The yields of pure protein varied markedly, from 40 mg pure protein/L of culture for WT-β_2_m (Asp76) to 0.4 mg pure protein/L of culture for D76L-β_2_m (*SI Appendix*, Table S3). The apparent thermal stability (T_m;app_) of each protein measured by far-UV CD (far ultra-violet circular dichroism) also varied markedly, from 65.2 ± 0.4 °C for WT-β_2_m to 37.9 ± 0.8 °C for D76R-β_2_m (Fig. 4*A*; see also *SI Appendix*, Fig. S6 and Table S3). This variation is surprising, given that the sidechain of residue 76 is solvent exposed and makes just three hydrogen bonds (to the sidechains of N42, T73, and Y78) in the crystal structure of WT- and D76N-β_2_m (*SI Appendix*, Fig. S7 *A* and *E*). Nonetheless, a clear correlation (*r* = 86% using rank-based Spearman correlation; *SI Appendix*, Fig. S8 *A* and *B*) is observed between T_m;app_ and the in vivo growth score, suggesting that stability (as well as solubility) is an important parameter in determining the readout of the TPBLA for this series of variants (Fig. 3*D*). Finally, the aggregation rate of each variant was determined (monitored by thioflavin T [ThT] fluorescence) (*SI Appendix*, Fig. S6). Here, again, a wide variation in behavior was observed. As expected, WT-β_2_m did not form detectable amyloid fibrils under the conditions employed (40 μM protein, 25 mM sodium phosphate buffer, 115 mM NaCl, pH 6.2, 37 °C, and shaking), while D76N-β_2_m formed amyloid-like fibrils the most rapidly of all 20 variants under the conditions employed (T_half_ 9.6 ± 3.8 h) (*SI Appendix*, Table S3). Of the remaining variants, nine (D76A-, D76T-, D76S-, D76V-, D76Q-, D76G-, D76H-, D76M-, and D76K-β_2_m) formed amyloid-like fibrils rapidly (T_half_ < 30 h; below the lower dashed line in Fig. 4*B*), while the other seven either failed to form fibrils (D76Y-, D76L-, D76I-, D76W-, D76F-, D76C-, and D76R-β_2_m; above the higher dashed line in Fig. 4*B*) or formed fibrils very slowly (T_half_ 60 to 70 h for D76E- and D76P-β_2_m; Fig. 4*B* and *SI Appendix*, Table S3). Indeed, there is only a poor correlation between the in vivo growth score and the T_half_ value (*r* = 28% using rank-based Spearman correlation; *SI Appendix*, Fig. S8 *C* and *D*), with the four variants with the lowest in vivo growth score (worst behavior; D76W-, D76F-, D76C-, and D76R-β_2_m) failing to aggregate in vitro under the conditions employed. D76N-, D76Q-, and D76M-β_2_m, which have intermediate in vivo growth scores, aggregate the most rapidly (Fig. 4*B*). Comparing protein stability and aggregation (Fig. 4 *A* and *B*) suggests that variants that are most (T_m;app_ > 60 °C; WT-β_2_m) or least stable (T_m;app_ ≤ 48 °C; D76L-, D76P-, D76I-, D76W-, D76F-, D76C-, and D76R-β_2_m) (Fig. 4*A*) either lose the ability to form amyloid fibrils or do so very slowly (with the sole exception of D76M-β_2_m: T_m;app_ 48.7 ± 0.4 °C and T_half_ 10.3 ± 1.2 h). This observation indicates that a “sweet spot” for β_2_m aggregation involves residue 76 in that a highly stable native protein does not aggregate, presumably since it is “trapped” in the native state, while for destabilized variants, the ability to unfold provides the potential to aggregate, depending on the precise amino acid sequence of the unfolded state.

### Structural Differences of D76X-β_2_m Variants.

Le Marchand et al. recently hypothesized that changes in the hydrogen bond network involving Asp-76 of WT-β_2_m could play a role in determining the uniquely high aggregation propensity of D76N-β_2_m (39). To examine the structural effect of other amino acid substitutions at this site, the crystal structures of seven variants (D76E-, D76A-, D76S-, D76G-, D76Q-, D76Y-, and D76K-β_2_m) were determined at high resolution (1.15 to 1.52 Å) (*SI Appendix*, Fig. S7 and Table S4). Six variants could not be crystallized or yielded crystals too small to be fished (D76H-, D76C-, D76I-, D76W-, D76T-, and D76V-β_2_m), and the yield of the remaining five proteins was too low to enable crystallization trials. Comparison of the crystal structures of D76E-, D76A-, D76S-, D76G-, D76Q-, D76Y-, and D76K-β_2_m with those of WT- and D76N-β_2_m revealed no substantial differences that could explain their different stabilities and aggregation propensities (RMSD < 0.6 Å over all heavy atoms; *SI Appendix*, Table S5 and Fig. 4*C*). In WT-β_2_m, a salt bridge is formed between the negatively charged residue Asp-76 and the highly conserved Lys41. This interaction is preserved in D76E-β_2_m (*SI Appendix*, Fig. S7*B*) and may explain, at least in part, the protection of β_2_m from aggregation for these two proteins (Fig. 3*A*). Indeed, a recent study has highlighted the importance of this salt bridge for D76N-β_2_m stability and aggregation (47). Interestingly, two of the most rapidly aggregating variants (D76N- and D76Q-β_2_m) share an amide sidechain. For D76N-β_2_m, the network of hydrogen bonds involving the sidechains of N42, T73, and Y78 found in the CD and EF loops of WT-β_2_m is maintained in the variant (*SI Appendix*, Fig. S7 *A* and *E*). By contrast, the sidechain of Gln76 moves away from Asn42 in the variant D76Q-β_2_m; instead, Gln76 hydrogen bonds with Lys41 (*SI Appendix*, Fig. S7 *E* and *F*), similar to D76E-β_2_m (*SI Appendix*, Fig. S7*B*). For other variants, the loop structure is maintained despite these hydrogen bonds either not forming (D76A-, D76G-, and D76Y-β_2_m; *SI Appendix*, Fig. S7 *C*, *G*, and *H*, respectively), or being reduced in number (D76K-β_2_m hydrogen bonds to N42, and D76S-β_2_m hydrogen bonds to N42 and T73; *SI Appendix*, Fig. S7 *I* and *D*, respectively). Together, these observations indicate that the sidechain chemistry at residue 76 plays a role in protein stability and aggregation, but the behavior of the variants cannot be rationalized by their crystal structures alone.

### Evolving D76N-β_2_m to Reduce Its Aggregation Propensity.

**Fig. 5.**
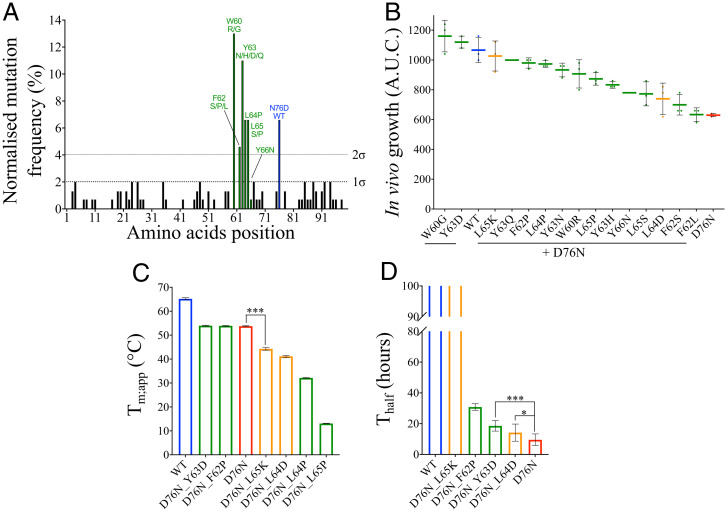
Evolving D76N-β_2_m to improve its properties. (*A*) Frequency of amino acid substitutions for the 56 unique sequences during screening of the D76N-β_2_m* library reveals the APR (residues 60 to 66, in green), with five of these residues having a mutational frequency higher than 1σ (residues 60, 62, 63, 64, and 65). Residue 76 (in blue) corresponds to the substitution that restores WT-β_2_m. Residue 66 was the only residue with a mutational frequency lower than 1σ to have a positive effect on D76N-β_2_m. (*B*) In vivo growth scores of 15 variants (D76N + X) compared with WT-β_2_m and D76N-β_2_m. In total, 13 variants were found in the APR (*A*, in green), and two variants were selected using Solubis (in orange; *SI Appendix*, Fig. S12). Data represent the mean (*n* = three biologically independent repeats), where each point corresponds to one experiment. The variants are ordered from the highest to lowest in vivo growth score (AUC) (*Left* to *Righ*t). The error bar represents one SD. Note that a larger range of ampicillin concentration was used to determine the behavior of these improved variants (0 to 280 μg mL^−1^) (*SI Appendix*, *Materials and Methods*); hence, the AUC is greater than those shown for D76N-β_2_m and WT-β_2_m in Figs. 1*D* and 3. (*C*) Protein stability (T_m;app_) for WT-β_2_m, D76N-β_2_m, and six variants containing amino acid changes in the APR (i.e., double variants D76N + X, where X is a residue in the APR) obtained using temperature ramp monitored by far-UV CD. The error bar is the fitting error. (*D*) Protein aggregation rate (T_half_) for WT-β_2_m, D76N-β_2_m, and four of the double variants, determined using ThT fluorescence. The error bar represents one SD of 8 to 10 repeats. Note that insufficient D76N_L64P- and D76N_L65P-β_2_m could be purified to perform the aggregation assay. Asterisks denote significance (*t* test: paired two sample for means, two-tail) where *P = 0.04 and ***P < 0.0001. Note that the same color code is used for *B*–*D*.

Having established that the TPBLA can be used to differentiate the effects of amino acid substitutions at positions 53, 76, and 98 in β_2_m, we next sought to use the assay as a screen for directed evolution to select for D76N-β_2_m variants with improved properties, selected by an increased in vivo growth score. Genetic variation was introduced into the gene encoding D76N-β_2_m using error-prone PCR before inserting the resulting library (βLa-D76N-β_2_m*) into the β-lac vector (*SI Appendix*, *Materials and Methods*). The DNA sequences of 22 clones in the naïve library revealed an average mutational frequency of 10.9 base pair (b.p.) mutations per 1,000 b.p. (corresponding to 3.2 b.p. mutations per D76N-β_2_m gene, equivalent to 2.4 amino acid substitutions per protein sequence). To screen for variants with enhanced properties, the βLa-D76N-β_2_m* plasmid library was transformed into *E. coli* SCS1 cells, and the bacteria were plated onto agar containing 120 or 140 µg mL^−1^ of ampicillin. In total, 1,000 and 100 colonies, respectively, were observed after transformation with βLa-D76N-β_2_m*. Transformation with the undiversified βLa-D76N-β_2_m plasmid yielded five and zero colonies at 120 or 140 µg mL^−1^ of ampicillin, respectively, suggesting that some mutations in βLa-D76N-β_2_m* increase the expression of functional βLa-β_2_m. DNA sequencing of 209 colonies selected randomly at these two antibiotic concentrations yielded 56 unique sequences involving amino acid substitutions at 52 different sites that are scattered throughout the protein sequence, including residues in the β-strands and their connecting loops (*SI Appendix*, Fig. S9*A*). Ten of these unique sequences result in an amino acid substitution at residue 76, of which nine contain the WT reversion substitution (N76D), either in isolation or in combination with either one or two additional amino acid changes, while one contained the substitution N76S in combination with a mutation in the APR predicted by Tango (18) (W60R) (*SI Appendix*, Table S6). Interestingly, for the 46 unique sequences that did not involve substitution of residue 76, all contained one or more amino acid changes in the region spanning residues 60 to 66, which contains the loop linking β-strands D and E (the DE loop) and β-strand E in the native structure (Fig. 5*A* and *SI Appendix*, Table S7 and Fig. S9*A*). Notably, this region is the single most aggregation-prone region of the sequence predicted by Tango (18) (Fig. 2*H*) and has been shown previously to be highly aggregation prone in isolation (48). Consistent with this, all but one of the sequences were predicted by Solubis (21, 22) to decrease the aggregation propensity (ΔTango) or to decrease stability (ΔΔG°), determined using FoldX (17) (*SI Appendix*, Fig. S10*A*).

The in vivo growth score was measured for the 46 unique sequence variants (excluding the 10 sequences that contain a substitution at residue 76), and each was compared with the score for WT- and D76N-β_2_m. All 46 of the evolved variants displayed an enhanced in vivo growth score relative to D76N-β_2_m, and 22 variants had a score that exceeds that of WT-β_2_m (*SI Appendix*, Fig. S10*B*). All these sequences have at least one amino acid substitution in the region spanning residues 60 to 66, which spans the APR defined by Tango (predicted to span residues 60 to 68) (Fig. 2*H*). Residue 59 was not altered in our screen. Residue 66 was substituted in only one unique sequence (in combination with F22L and K48E [D76N_F22L-K48E-Y66N-β_2_m]), while residue 67 was found in two unique sequences but always in combination with either an amino acid substitution at residues 63 or 64 (*SI Appendix*, Table S7). This suggests that substitutions at F22 and K48 may be passively selected due to the presence of additional beneficial mutations in the APR. Consistent with this, analysis of the D76N_F22L- and D76N_K48E-β_2_m variants individually using the TPBLA yielded proteins with slightly reduced *in vivo* growth scores compared with D76N-β_2_m, indicating that individual alteration of these three residues has no beneficial effect on D76N-β_2_m (*SI Appendix*, Fig. S9*B*). We, thus, define the APR of D76N-β_2_m as spanning residues 60 to 66 (^60^WSFYLLY^66^).

In all, 13 different amino acid substitutions in the APR (residues 60 to 66, ^60^WSFYLLY^66^) were found either singly, in combination with other substitutions in the APR or elsewhere, or both (*SI Appendix*, Table S7). These 13 single variants were cloned into βLa-D76N-β_2_m, and their in vivo growth scores were determined using the TPBLA. All increased the in vivo growth score of D76N-β_2_m (colored green for residues 60 to 66 in Fig. 5*B*), with two substitutions (W60G and Y63D) giving scores higher than WT-β_2_m (Fig. 5*B*). This highlights a key role of the APR in defining the properties of D76N-β_2_m in the TPBLA. Notably, the best-scoring substitution of all evolved variants was W60G (Fig. 5*B*). This solvent-exposed tryptophan lies in the DE loop of native β_2_m and is required for its function (49). Substitution with glycine has been shown to stabilize the native protein and to reduce its aggregation propensity (49, 50). Instead of glycine, arginine was found in four of the nine variants substituted for W60. This accords with previous work showing that either W60R or W60C also reduces aggregation propensity (39, 51).

To confirm the importance of residues 60 to 66 in determining the behavior of D76N-β_2_m in the TPBLA, Solubis (21, 22) was used to design two single-residue substitutions in the APR that are each predicted to significantly reduce the aggregation propensity of D76N-β_2_m (L64D ΔTango −750.9 and L65K ΔTango −560.0). These substitutions are also predicted to destabilize the protein (ΔΔG° 4.70 and 1.17 kcal mol^−1^ for L64D and L65K, respectively, calculated using FoldX; ref. 17). As predicted, the introduction of a negatively charged (Asp) or positively charged (Lys) residue into the center of the APR (at residue 64 or 65, respectively) each improved the in vivo growth score of D76N-β_2_m (colored orange in Fig. 5*B*). While L64D only slightly (but significantly) improves the in vivo growth score of D76N-β_2_m, the substitution L65K improves the score dramatically, such that this variant behaves similarly to WT-β_2_m. Notably, no correlation is observed between the in vivo growth score and the predictors Tango (18), CamSol (16), or Aggrescan 3D (23, 24) (*r* < 25% using rank-based Spearman correlation; *SI Appendix*, Fig. S11), consistent with the complexity in factors that determines the behavior of D76N-β_2_m variant in the TPBLA.

The APR of β_2_m, ^60^WSFYLLY^66^, is composed mainly of aromatic and hydrophobic residues, consistent with its high predicted Tango score (18) (Fig. 2*H*) and measured aggregation propensity (as an isolated peptide; ref. 48). All amino acid substitutions resulting from the error-prone PCR decrease the hydrophobicity of the APR by substitution with a polar or charged residue (Lys, His, Arg, Asp, Ser, Asn, or Gln), by reducing the size of the sidechain (e.g., Phe to Leu or Gly), or by incorporation of the β-strand breaking residue Pro. Consistent with this, analysis of the properties of the 46 unique sequences using Solubis (21, 22) predicts that 45 of the 46 variants have reduced aggregation propensity compared to WT- and D76N-β_2_m (the sole exception is D76N_F62L-H84R-W95R-β_2_m [clone 27], which is the only substitution in the APR that maintains a hydrophobic residue [other than substitution with Pro]; *SI Appendix*, Fig. S10*A*). Most of these variants (44 of the 46 unique sequences) have a concomitant predicted decrease in stability (ΔΔG° > 1 kcal mol^−1^; *SI Appendix*, Fig. S10*A*) (with the exception of D76N_W60R-β_2_m [clone 9] and D76N_S57G-W60R-β_2_m [clone 11], which only slightly decrease the predicted aggregation propensity; ΔTango ∼ −130), consistent with previous results suggesting that reducing aggregation by sequence alteration generally comes at the cost of reducing protein stability (52).

Finally, to explore whether amino acid substitutions at residues outside of the APR could decrease aggregation—even though they were not observed here experimentally—saturation mutagenesis of every residue in D76N-β_2_m was performed in silico using Solubis (21, 22) (*SI Appendix*, Fig. S12). Consistent with the single dominant APR in the β_2_m sequence, these results showed that of the 1,900 possible amino acid substitutions, the only substitutions that decrease aggregation (53 single-point variants for which ΔTango > −200; *SI Appendix*, Fig. S12) involve residues in the APR (with the sole exception of Y67P, ΔTango −303). Of these 53 single-point variants, 89% are predicted to be destabilized (ΔΔG° > 1 kcal mol^−1^) relative to D76N-β_2_m, while only six variants (11%) either have no effect on stability (D76N_L65E-, D76N_L65R-, D76N_Y63K-, D76N_W60E-, and D76N_S61D-β_2_m; ΔΔG° < 1 kcal mol^−1^) or marginally stabilize D76N-β_2_m (D76N_S61E-β_2_m; ΔΔG° −0.85 kcal mol^−1^) (*SI Appendix*, Fig. S12).

### Purified Proteins with Sequence Substitutions in the APR Have Decreased Stability and Decreased Aggregation Propensity.

To determine how sequence alterations in the APR of D76N-β_2_m affect protein stability and aggregation propensity, six of the variants with an enhanced in vivo growth score were cloned, expressed, and purified, creating the proteins D76N_F62P-, D76N_Y63D-, D76N_L64D-, D76N_L64P-, D76N_L65P-, and D76N_L65K-β_2_m. These variants span the APR and include the four sequence substitutions with the highest in vivo growth scores at each site from the TPBLA, along with the two designed variants, D76N_L64D- and D76N_L65K-β_2_m, which were predicted to be highly aggregation resilient but not severely destabilized using Solubis (*SI Appendix*, Fig. S12). The stability (T_m;app_) for the six variants was determined, while only four of the variants could be purified in an amount sufficient for analysis of their aggregation rate (T_half_) (D76N_L65K-, D76N_F62P-, D76N_Y63D-, and D76N_L64D-β_2_m) (*SI Appendix*, *Materials and Methods*; Fig. 5 *C* and *D* and *SI Appendix*, Table S8 and Fig. S13). Surprisingly, and in contrast with predictions based on Solubis (21, 22) (*SI Appendix*, Fig. S12) and previous predictions (52)—which suggest, in general, that reducing aggregation comes with the cost of destabilizing a protein—several counterexamples were found for D76N-β_2_m. For example, using Solubis, D76N_L64D-β_2_m is predicted to abolish the APR (21, 22) (*SI Appendix*, Fig. S12) by adding a gatekeeper residue in the middle of the APR (cutting the APR in two), but the aggregation rate (T_half_) of this variant is only marginally longer than that of D76N-β_2_m (Fig. 5*D* and *SI Appendix*, Fig. S13*C*). Moreover, D76N_F62P- and D76N_Y63D-β_2_m have similar stability as D76N-β_2_m (Fig. 5*C* and *SI Appendix*, Fig. S13 *A* and *B*), yet Solubis predicts these mutations to be destabilizing (*SI Appendix*, Fig. S12). Finally, only one mutation (L65K) was found to abolish aggregation (Fig. 5*C* and *SI Appendix*, Fig. S13*D*), in agreement with Solubis (21, 22) (*SI Appendix*, Fig. S12). These results highlight the complexity of predicting the effect of a mutation on aggregation, which we show depends on a critical balance of native-state stability, solubility, the presence of the APR in the sequence, and the properties of nonnative states that are formed at different stages of the aggregation cascade (*SI Appendix*, Fig. S14).

Finally, to determine whether amino acid substitutions in the APR of D76N-β_2_m have a similar effect on the WT protein, the six sequence alterations in the APR of D76N-β_2_m discussed above were introduced into WT-β_2_m, and the in vivo growth score of each was determined using the TPBLA. Strikingly, the results revealed that the effects of the amino acid substitutions are different for WT- and D76N-β_2_m, dependent on the site of substitution and the identity of the residue introduced (*SI Appendix*, Fig. S15). In WT-β_2_m, three of the variants in the APR increased the *in vivo* growth score (F62P, Y63D, and L65K), one had little effect (L64D), and two reduced the score (L64P and L65P). By contrast, all six variants increased the in vivo growth score of D76N-β_2_m. This suggests a difference in the stability/aggregation tradeoff and/or differences in the mechanisms of aggregation of the two proteins, despite the fact that the proteins differ by only a single amino acid substitution in a solvent-exposed loop that is distant in sequence and space to residues in the APR.

### Natural Evolution of β_2_m Sequences.

We next wondered whether the APR of β_2_m is conserved among species, especially given that this sequence appears to drive aggregation of D76N-β_2_m. Analysis of 262 sequences of β_2_m from animals throughout Mammalia showed that residues in the APR (particularly Trp60, Phe62, Tyr63, Leu64, and Leu65) and Asp76 are highly conserved (*SI Appendix*, Fig. S16 *A*–*C*). With 100% identity across all 262 sequences, Trp60 and Phe62 form key contacts with the MHC 1 heavy chain, which is vital for its function (34). Leu64 (88% identity) forms part of the hydrophobic core of the protein, suggesting that this residue may be beneficial for stability, while Leu65 (100% conserved) is solvent exposed and may be important for folding rather than for stability or function. A total of 16 amino acid substitutions were found in the APR in these 262 sequences (0 substitutions at positions 60, 62, and 65; three substitutions at position 61; four substitutions at position 63; and five substitutions at positions 64 and 66). As we show above, residue 76 plays a unique role in determining the aggregation of human β_2_m (Fig. 3). Position 76 is Asp in 96% of the 262 mammalian sequences of β_2_m (*SI Appendix*, Fig. S16*C*). Six substitutions are observed at residue 76 (Ala, His, Asn, Ser, Thr, and Val). Interestingly, saturation mutagenesis of residue 76 showed that these six substitutions are among those that have the least effect on the in vivo growth score and on protein stability compared with Asp76 (Figs. 3*A* and 4*A*).

One β_2_m sequence was found (European rabbit) that contains an Asn at position 76 and retains a strong APR involving residues 60 to 66 (^60^WSFYLLV^66^, which differs from human β_2_m only at residue 66). To determine whether rabbit-β_2_m is as aggregation prone as its sequence might predict, the protein was expressed and purified, and its rate of aggregation, stability, and in vivo growth score were determined. Surprisingly, the results revealed that rabbit-β_2_m is more resilient to aggregation than human D76N-β_2_m, with a high in vivo growth score, a similar T_m;app_ to human D76N-β_2_m, and little or no aggregation over 100 h (*SI Appendix*, Fig. S16 *D*–*F*). Thus, even with an Asn at position 76 and a strong APR, the sequence of rabbit β_2_m must have evolved other means of preventing aggregation.

## Discussion

The TPBLA has been used previously to select for proteins with increased kinetic and thermodynamic stability (29, 30); to rank the aggregation propensity of amyloidogenic peptides (28); to screen for small-molecule inhibitors of protein aggregation (28); and, most recently, to evolve antibody fragments for increased aggregation resistance (27). Here, we have exploited the powers of the TPBLA to investigate the relationship among sequence, stability, and aggregation of the amyloidogenic protein D76N-β_2_m. This protein was selected for our study as its aggregation mechanism is complex, commencing from an initially stably structured protein with an all-antiparallel immunoglobulin fold, which presumably must reorganize substantially during formation of the cross-β structure of amyloid (4). The protein has also been shown to differ in its aggregation mechanism from WT-β_2_m (31, 39), despite the proteins differing by only a single amino acid in a solvent-exposed loop (15). Finally, previous studies of aggregation of WT-β_2_m under acidic conditions have shown that there is no relationship between aggregation rate and thermodynamic stability (36), suggesting that the high aggregation propensity of D76N-β_2_m cannot be rationalized by the protein’s reduced global stability alone.

The results presented here highlight the ability of the TPBLA to differentiate the behavior of β_2_m variants that differ by only a single residue, opening the door to the use of the assay to select for D76N-β_2_m sequence variants with enhanced properties. Firstly, using saturation mutagenesis at three different positions that each target a solvent-exposed Asp (residues 53, 76, and 98), we used the TPBLA to reveal the unique importance of residue 76 in defining the aggregation of β_2_m. The native Asp was most protective, Glu and Ala showed some protection, and all other residues (including Asn) resulted in a low in vivo growth score when introduced at this site. In marked contrast, there is no effect of amino acid substitution at residue 98, while residue 53 revealed behavior that is entirely consistent with its location in an edge strand (46).

The potential of a folded protein to assemble into amyloid fibrils depends on a complex combination of interdependent characteristics, each of which is affected by the protein sequence. These include properties of the native protein, such as thermodynamic and kinetic stability, solubility, cooperativity of the native fold, and the solvent accessibility of its usually buried APR(s) (52). The properties of the protein sequence—such as its inherent solubility; β-sheet propensity; the presence of APRs; and the type of residues that flank an APR, such as gatekeeper residues (Glu, Lys, Arg, Asp, and Pro) that suppress aggregation—also play a key role in determining whether a protein aggregates under a defined set of solution conditions (45). Aggregation may also be initiated from the native state; a structured, but nonnative, conformation (such as the I_T_-state for WT-β_2_m; ref. 41); the unfolded state; or a combination of these different species. As a consequence, predicting whether a folded protein will self-assemble into amyloid fibrils under a defined set of conditions remains challenging (3). Here, using the TPBLA to evolve sequence variants of D76N-β_2_m with higher in vivo growth scores, we show that the assay detects the “limiting factor” or “Achilles heel” of the protein sequence (i.e., the property that is least evolved and places the protein at the threshold of aggregation). Such residues make the protein hypersensitive to sequence alterations at that site. Saturation mutagenesis revealed that the in vivo growth score of β_2_m is sensitive to the identity of residue 76, with two other solvent-exposed sites in the protein (residues 53 and 98) displaying strikingly different behavior. The results also demonstrate the power of the TPBLA to reveal hotspots in a protein sequence that may cause its aggregation/insolubility and to select for sequence variants at different sites with improved properties. For the 20 D76X-β_2_m variants, a “sweet spot” between protein stability and aggregation was revealed, with the D76N variant aggregating rapidly in vitro, while proteins that are destabilized or stabilized relative to D76N-β_2_m aggregate more slowly, at least under the conditions explored here. Similar concepts of proteins at the “knife edge” have been described previously, in which protein stability and expression levels in vivo are critically balanced relative to their aggregation propensity (46, 53, 54).

In vivo evolution of D76N-β_2_m using the TPBLA confirmed the unique importance of residue 76 in determining the high aggregation propensity of this protein relative to WT-β_2_m, since sequences that revert Asn to the WT Asp were commonly detected (notably, the only other [one copy] substitution at this site was D76S; *SI Appendix*, Table S6). All other amino acid substitutions that improved the properties of D76N-β_2_m involved residues that lie in the APR (^60^WSFYLLY^66^), highlighting the dual importance of the identity of residue 76 and the APR in determining D76N-β_2_m aggregation. In silico analysis of the amino acid substitutions within the APR selected to improve the properties of D76N-β_2_m (using Solubis; ref. 21, 22) predict that 41% of the sequence changes that reduce the aggregation propensity of the APR (as judged by Tango; ref. 18) will also reduce protein stability (using FoldX; ref. 17) (*SI Appendix*, Fig. S12). This prediction is consistent with previous results that suggest that the presence of APRs is maintained in evolution since such regions stabilize a protein’s fold, but bring the associated cost that aggregation propensity is increased (52). In practice, however, the situation for D76N-β_2_m is more complex, with some substitutions in the APR having little effect on stability (e.g., F62P and Y63D), while others reduce stability (e.g., L64D, L64P, L65K, and L65P), and some reduce aggregation (e.g., F62P and L65K), while others have little effect (e.g., L64D) (Fig. 5 *C* and *D*).

Another striking finding from our study is that variants in the APR that enhance the properties of D76N-β_2_m have different effects on WT-β_2_m (*SI Appendix*, Fig. S15), consistent with the proteins aggregating by distinct molecular mechanisms despite differing by only a single residue (31). This suggests that residues in the APR that are important for aggregation must be enhanced by the presence of an Asn at residue 76, despite its distal location. This effect could be intramolecular (e.g., by affecting the propensity to expose the APR in nonnative monomers) or via Asn76 enhancing the population of on-pathway oligomers required for aggregation; *SI Appendix*, Fig. S14). Destabilization of kinetically trapped oligomers or the diversion of off-pathway oligomers toward more assembly-competent species would also give rise to the observed increased rate of amyloid formation of the D76N-β_2_m variant. Such mechanisms cannot be teased apart by the data presented here, and would require analysis of the effect of mutation on the structure and stability of oligomers and fibrils themselves (*SI Appendix*, Fig. S14). The findings that the amyloidogenicity of D76N-β_2_m cannot be rationalized by its reduced thermodynamic stability alone and that rabbit β_2_m does not aggregate in vitro under the conditions employed, despite having an Asn at residue 76, a highly conserved APR—and similar thermodynamic stability to D76N-β_2_m—highlights the importance of other residues in determining the amyloidogenicity of the protein via mechanisms that remain obscure.

Folded proteins that aggregate into amyloid can be divided into two classes: proteins that require global unfolding in order to initiate amyloid formation, as has been shown for antibody light chains (55, 56) and TTR (57); and proteins for which a specific partially folded species is required for aggregation to proceed (as shown for WT-β_2_m and ΔN6-β_2_m; ref. 41). The detailed molecular mechanism(s) by which the all-antiparallel β-sheet structure of native D76N-β_2_m is transformed into the cross-β structure of amyloid remain(s) unclear (31). One plausible mechanism could involve partial unfolding to a species that enables unzipping of the native β-strands and reorientation of the disulphide bond linking Cys25 to Cys80, akin to the mechanism proposed for amyloidogenic light chains (58). Since global stability, measured using thermal denaturation, does not correlate with the T_half_ of aggregation for the D76N-β_2_m variants analyzed here, global unfolding is an unlikely prerequisite of D76N-β_2_m aggregation. Previous results have shown that the formation of the structured I_T_-state is required to initiate the aggregation of WT- and ΔN6-β_2_m (41, 59, 60), yet amyloid formation of D76N-β_2_m does not depend on the formation of this state (31). Instead, we propose that one or more nonnative species that critically involve the APR and Asn76 are required to initiate amyloid formation of D76N-β_2_m. Such a model would rationalize the importance of both the APR and Asn76 for rapid aggregation, as well as the distinct aggregation mechanisms of the WT- and D76N-β_2_m variants. Further work will be needed to solve the structure of D76N-β_2_m amyloid fibrils and to explore the nature of the initiating steps in D76N-β_2_m aggregation so as to better understand how apparently innocuous sequence changes can have such a profound effect on the ability of this protein to aggregate into amyloid and cause disease.

## Materials and Methods

TPBLA assay, molecular biology, protein expression, protein purification, in vitro fibrillation assays, negative-stain EM (electron microscopy), thermal denaturation monitored by far-UV CD, equilibrium unfolding experiments monitored by fluorescence, prediction algorithms of stability solubility and amyloid propensity, crystallography, creation of the βLa-D76N-β_2_m library, directed evolution and selection of β_2_m variants using the TPBLA, prediction of the protein stability and protein aggregation using Solubis, and sequence alignment are described in detail in *SI Appendix*.

## Supplementary Material

Supplementary File

## Data Availability

All raw data for figures have been deposited to the University of Leeds DOI site (https://doi.org/10.5518/1073). All study data are included in the article and/or *SI Appendix* (62).

## References

[1] M. G. Iadanza, M. P. Jackson, E. W. Hewitt, N. A. Ranson, S. E. Radford, A new era for understanding amyloid structures and disease. Nat. Rev. Mol. Cell Biol. 19, 755–773 (2018). 30237470 10.1038/s41580-018-0060-8 PMC7617691

[2] J. Santos, J. Pujols, I. Pallarès, V. Iglesias, S. Ventura, Computational prediction of protein aggregation: Advances in proteomics, conformation-specific algorithms and biotechnological applications. Comput. Struct. Biotechnol. J. 18, 1403–1413 (2020). 32637039 10.1016/j.csbj.2020.05.026 PMC7322485

[3] J. S. Ebo, N. Guthertz, S. E. Radford, D. J. Brockwell, Using protein engineering to understand and modulate aggregation. Curr. Opin. Struct. Biol. 60, 157–166 (2020). 32087409 10.1016/j.sbi.2020.01.005 PMC7132541

[4] R. Gallardo, N. A. Ranson, S. E. Radford, Amyloid structures: Much more than just a cross-β fold. Curr. Opin. Struct. Biol. 60, 7–16 (2020). 31683043 10.1016/j.sbi.2019.09.001 PMC7617690

[5] S. Sakagashira , Missense mutation of amylin gene (S20G) in Japanese NIDDM patients. Diabetes 45, 1279–1281 (1996). 8772735 10.2337/diab.45.9.1279

[6] C. Nilsberth , The ‘Arctic’ APP mutation (E693G) causes Alzheimer’s disease by enhanced Abeta protofibril formation. Nat. Neurosci. 4, 887–893 (2001). 11528419 10.1038/nn0901-887

[7] C. Arber , Familial Alzheimer’s disease patient-derived neurons reveal distinct mutation-specific effects on amyloid beta. Mol. Psychiatry 25, 2919–2931 (2020). 30980041 10.1038/s41380-019-0410-8 PMC7577860

[8] Y. Sekijima, Transthyretin (ATTR) amyloidosis: Clinical spectrum, molecular pathogenesis and disease-modifying treatments. J. Neurol. Neurosurg. Psychiatry 86, 1036–1043 (2015). 25604431 10.1136/jnnp-2014-308724

[9] L. Jespers, O. Schon, K. Famm, G. Winter, Aggregation-resistant domain antibodies selected on phage by heat denaturation. Nat. Biotechnol. 22, 1161–1165 (2004). 15300256 10.1038/nbt1000

[10] G. Merlini , Systemic immunoglobulin light chain amyloidosis. Nat. Rev. Dis. Primers 4, 38 (2018). 30361521 10.1038/s41572-018-0034-3

[11] J. Floege , Clearance and synthesis rates of beta 2-microglobulin in patients undergoing hemodialysis and in normal subjects. J. Lab. Clin. Med. 118, 153–165 (1991). 1856578

[12] J. Kay, Beta 2-microglobulin amyloidosis in renal failure: Understanding this recently recognized condition. Cleve. Clin. J. Med. 66, 145–147 (1999). 10079583 10.3949/ccjm.66.3.145

[13] S. Otsubo , Characteristics of dialysis-related amyloidosis in patients on haemodialysis therapy for more than 30 years. Nephrol. Dial. Transplant. 24, 1593–1598 (2009). 19096084 10.1093/ndt/gfn706

[14] H. Mizuno , Dialysis-related amyloidosis associated with a novel β_2_-microglobulin variant. Amyloid 28, 42–49 (2021). 32875920 10.1080/13506129.2020.1813097

[15] S. Valleix , Hereditary systemic amyloidosis due to Asp76Asn variant β2-microglobulin. N. Engl. J. Med. 366, 2276–2283 (2012). 22693999 10.1056/NEJMoa1201356

[16] P. Sormanni, F. A. Aprile, M. Vendruscolo, The CamSol method of rational design of protein mutants with enhanced solubility. J. Mol. Biol. 427, 478–490 (2015). 25451785 10.1016/j.jmb.2014.09.026

[17] J. Van Durme , A graphical interface for the FoldX forcefield. Bioinformatics 27, 1711–1712 (2011). 21505037 10.1093/bioinformatics/btr254

[18] R. Linding, J. Schymkowitz, F. Rousseau, F. Diella, L. Serrano, A comparative study of the relationship between protein structure and beta-aggregation in globular and intrinsically disordered proteins. J. Mol. Biol. 342, 345–353 (2004). 15313629 10.1016/j.jmb.2004.06.088

[19] R. G. Parra , Protein frustratometer 2: A tool to localize energetic frustration in protein molecules, now with electrostatics. Nucleic Acids Res. 44 (W1), W356-60 (2016). 27131359 10.1093/nar/gkw304 PMC4987889

[20] A. O. Rausch , FrustratometeR: An R-package to compute local frustration in protein structures, point mutants and MD simulations. Bioinformatics 37, 3038–3040 (2021). 10.1093/bioinformatics/btab176 33720293

[21] J. Van Durme , Solubis: A webserver to reduce protein aggregation through mutation. Protein Eng. Des. Sel. 29, 285–289 (2016). 27284085 10.1093/protein/gzw019

[22] R. van der Kant, J. van Durme, F. Rousseau, J. Schymkowitz, SolubiS: Optimizing protein solubility by minimal point mutations. Methods Mol. Biol. 1873, 317–333 (2019). 30341620 10.1007/978-1-4939-8820-4_21

[23] A. Kuriata , Aggrescan3D (A3D) 2.0: Prediction and engineering of protein solubility. Nucleic Acids Res. 47 (W1), W300–W307 (2019). 31049593 10.1093/nar/gkz321 PMC6602499

[24] R. Zambrano , AGGRESCAN3D (A3D): Server for prediction of aggregation properties of protein structures. Nucleic Acids Res. 43 (W1), W306-13 (2015). 25883144 10.1093/nar/gkv359 PMC4489226

[25] I. Pallarès, S. Ventura, Understanding and predicting protein misfolding and aggregation: Insights from proteomics. Proteomics 16, 2570–2581 (2016). 27479752 10.1002/pmic.201500529

[26] M. E. Cromwell, E. Hilario, F. Jacobson, Protein aggregation and bioprocessing. AAPS J. 8, E572–E579 (2006). 17025275 10.1208/aapsj080366 PMC2761064

[27] J. S. Ebo , An in vivo platform to select and evolve aggregation-resistant proteins. Nat. Commun. 11, 1816 (2020). 32286330 10.1038/s41467-020-15667-1 PMC7156504

[28] J. C. Saunders , An in vivo platform for identifying inhibitors of protein aggregation. Nat. Chem. Biol. 12, 94–101 (2016). 26656088 10.1038/nchembio.1988 PMC4720988

[29] T. T. Hailu, L. Foit, J. C. Bardwell, In vivo detection and quantification of chemicals that enhance protein stability. Anal. Biochem. 434, 181–186 (2013). 23219982 10.1016/j.ab.2012.11.022 PMC3670414

[30] L. Foit , Optimizing protein stability in vivo. Mol. Cell 36, 861–871 (2009). 20005848 10.1016/j.molcel.2009.11.022 PMC2818778

[31] H. I. Smith , The role of the I_T_-state in D76N β_2_-microglobulin amyloid assembly: A crucial intermediate or an innocuous bystander? J. Biol. Chem. 295, 12474–12484 (2020). 32661194 10.1074/jbc.RA120.014901 PMC7458819

[32] D. E. Isenman, R. H. Painter, K. J. Dorrington, The structure and function of immunoglobulin domains: Studies with beta-2-microglobulin on the role of the intrachain disulfide bond. Proc. Natl. Acad. Sci. U.S.A. 72, 548–552 (1975). 47633 10.1073/pnas.72.2.548 PMC432350

[33] E. C. Campbell, A. N. Antoniou, S. J. Powis, The multi-faceted nature of HLA class I dimer molecules. Immunology 136, 380–384 (2012). 22533699 10.1111/j.1365-2567.2012.03593.x PMC3401976

[34] P. Cresswell, A. L. Ackerman, A. Giodini, D. R. Peaper, P. A. Wearsch, Mechanisms of MHC class I-restricted antigen processing and cross-presentation. Immunol. Rev. 207, 145–157 (2005). 16181333 10.1111/j.0105-2896.2005.00316.x

[35] R. N. Germain, Immunology. The ins and outs of antigen processing and presentation. Nature 322, 687–689 (1986). 3489186 10.1038/322687a0

[36] S. L. Myers , A systematic study of the effect of physiological factors on beta2-microglobulin amyloid formation at neutral pH. Biochemistry 45, 2311–2321 (2006). 16475820 10.1021/bi052434i PMC7618240

[37] M. de Rosa , Decoding the structural bases of D76N beta2-microglobulin high amyloidogenicity through crystallography and Asn-Scan mutagenesis. PLoS One 10, e0144061 (2015). 26625273 10.1371/journal.pone.0144061 PMC4666650

[38] G. Esposito , Removal of the N-terminal hexapeptide from human beta2-microglobulin facilitates protein aggregation and fibril formation. Protein Sci. 9, 831–845 (2000). 10850793 10.1110/ps.9.5.831 PMC2144642

[39] T. Le Marchand , Conformational dynamics in crystals reveal the molecular bases for D76N beta-2 microglobulin aggregation propensity. Nat. Commun. 9, 1658 (2018). 29695721 10.1038/s41467-018-04078-y PMC5916882

[40] K. E. Routledge, G. G. Tartaglia, G. W. Platt, M. Vendruscolo, S. E. Radford, Competition between intramolecular and intermolecular interactions in an amyloid-forming protein. J. Mol. Biol. 389, 776–786 (2009). 19393661 10.1016/j.jmb.2009.04.042 PMC2722902

[41] T. K. Karamanos , Structural mapping of oligomeric intermediates in an amyloid assembly pathway. eLife 8, 46574 (2019). 10.7554/eLife.46574 PMC6783270 31552823

[42] T. K. Karamanos , A population shift between sparsely populated folding intermediates fetermines amyloidogenicity. J. Am. Chem. Soc. 138, 6271–6280 (2016). 27117876 10.1021/jacs.6b02464 PMC4922733

[43] E. Rennella, G. J. Morgan, N. Yan, J. W. Kelly, L. E. Kay, The role of protein thermodynamics and primary structure in fibrillogenesis of variable domains from immunoglobulin light chains. J. Am. Chem. Soc. 141, 13562–13571 (2019). 31364359 10.1021/jacs.9b05499 PMC6850217

[44] P. Sormanni, L. Amery, S. Ekizoglou, M. Vendruscolo, B. Popovic, Rapid and accurate in silico solubility screening of a monoclonal antibody library. Sci. Rep. 7, 8200 (2017). 28811609 10.1038/s41598-017-07800-w PMC5558012

[45] B. Houben , Autonomous aggregation suppression by acidic residues explains why chaperones favour basic residues. EMBO J. 39, e102864 (2020). 32237079 10.15252/embj.2019102864 PMC7265246

[46] J. S. Richardson, D. C. Richardson, Natural beta-sheet proteins use negative design to avoid edge-to-edge aggregation. Proc. Natl. Acad. Sci. U.S.A. 99, 2754–2759 (2002). 11880627 10.1073/pnas.052706099 PMC122420

[47] É. Bulyáki , Pathogenic D76N variant of β_2_-microglobulin: Synergy of diverse effects in both the native and amyloid states. Biology (Basel) 10, 1197 (2021). 34827190 10.3390/biology10111197 PMC8614874

[48] S. Jones, J. Manning, N. M. Kad, S. E. Radford, Amyloid-forming peptides from beta2-microglobulin-insights into the mechanism of fibril formation in vitro. J. Mol. Biol. 325, 249–257 (2003). 12488093 10.1016/s0022-2836(02)01227-5

[49] G. Esposito , The controlling roles of Trp60 and Trp95 in beta2-microglobulin function, folding and amyloid aggregation properties. J. Mol. Biol. 378, 887–897 (2008). 18395224 10.1016/j.jmb.2008.03.002

[50] C. Camilloni , Rational design of mutations that change the aggregation rate of a protein while maintaining its native structure and stability. Sci. Rep. 6, 25559 (2016). 27150430 10.1038/srep25559 PMC4858664

[51] S. Ricagno , DE loop mutations affect beta2-microglobulin stability and amyloid aggregation. Biochem. Biophys. Res. Commun. 377, 146–150 (2008). 18835253 10.1016/j.bbrc.2008.09.108

[52] T. Langenberg , Thermodynamic and evolutionary coupling between the native and amyloid state of globular proteins. Cell Rep. 31, 107512 (2020). 32294448 10.1016/j.celrep.2020.03.076 PMC7175379

[53] J. Kirstein-Miles, A. Scior, E. Deuerling, R. I. Morimoto, The nascent polypeptide-associated complex is a key regulator of proteostasis. EMBO J. 32, 1451–1468 (2013). 23604074 10.1038/emboj.2013.87 PMC3655472

[54] G. G. Tartaglia, S. Pechmann, C. M. Dobson, M. Vendruscolo, Life on the edge: A link between gene expression levels and aggregation rates of human proteins. Trends Biochem. Sci. 32, 204–206 (2007). 17419062 10.1016/j.tibs.2007.03.005

[55] L. Radamaker , Cryo-EM structure of a light chain-derived amyloid fibril from a patient with systemic AL amyloidosis. Nat. Commun. 10, 1103 (2019). 30894526 10.1038/s41467-019-09032-0 PMC6427026

[56] A. Schmidt, K. Annamalai, M. Schmidt, N. Grigorieff, M. Fändrich, Cryo-EM reveals the steric zipper structure of a light chain-derived amyloid fibril. Proc. Natl. Acad. Sci. U.S.A. 113, 6200–6205 (2016). 27185936 10.1073/pnas.1522282113 PMC4896715

[57] L. Saelices , Uncovering the mechanism of aggregation of human transthyretin. J. Biol. Chem. 290, 28932–28943 (2015). 26459562 10.1074/jbc.M115.659912 PMC4661406

[58] M. G. Iadanza , The structure of a β_2_-microglobulin fibril suggests a molecular basis for its amyloid polymorphism. Nat. Commun. 9, 4517 (2018). 30375379 10.1038/s41467-018-06761-6 PMC6207761

[59] T. R. Jahn, M. J. Parker, S. W. Homans, S. E. Radford, Amyloid formation under physiological conditions proceeds via a native-like folding intermediate. Nat. Struct. Mol. Biol. 13, 195–201 (2006).1649109210.1038/nsmb1058

[60] M. Sakata , Kinetic coupling of folding and prolyl isomerization of beta2-microglobulin studied by mutational analysis. J. Mol. Biol. 382, 1242–1255 (2008). 18708068 10.1016/j.jmb.2008.08.003

[61] C. H. Trinh, D. P. Smith, A. P. Kalverda, S. E. Phillips, S. E. Radford, Crystal structure of monomeric human beta-2-microglobulin reveals clues to its amyloidogenic properties. Proc. Natl. Acad. Sci. U.S.A. 99, 9771–9776 (2002). 12119416 10.1073/pnas.152337399 PMC125010

[62] N. Guthertz ., Dataset associated with “The effect of mutation on an aggregation-prone protein: An in vivo, in vitro and in silico analysis.” Dataset. 10.5518/1073. Deposited 11 November 2021.

