# Supplementary material for: The effect of mutation on an aggregation-prone protein: An in vivo, in vitro, and in silico analysis
Source: Proc Natl Acad Sci U S A. 2022 May 25;119(22):e2200468119. doi: 10.1073/pnas.2200468119 (PMC9295795; doi:10.1073/pnas.2200468119)
Supplement: Supplementary File [file pnas.2200468119.sapp.pdf]

SUPPLEMENTARTY APPENDIX

The effect of mutation on an aggregation-prone protein: An *in vivo, in vitro* and *in silico* analysis

N. Guthertz[1], R. van der Kant[2,3], R. M. Martinez[1], Y. Xu[1], C. Trinh[1], B. I. Iorga[4], F. Rousseau[2,3], J. Schymkowitz[2,3], D. J. Brockwell[1], S. E. Radford[1,*]

[1] *Astbury Centre for Structural Molecular Biology, School of Molecular & Cellular Biology, Faculty of Biological Sciences, University of Leeds, Leeds, LS2 9JT, UK.*

[2] *Switch Laboratory, VIB-KU Leuven Center for Brain & Disease Research, Herestraat 49, 3000 Leuven, Belgium*

[3] *Department of Cellular and Molecular Medicine, KU Leuven, Herestraat 49, Box 802, 3000 Leuven, Belgium*

[4] *Université Paris-Saclay, CNRS UPR 2301, Institut de Chimie des Substances Naturelles, 91198 Gif-sur-Yvette, France*

* Corresponding author. Email: S.E.Radford@leeds.ac.uk (S. E. R.)

MATERIALS AND METHODS

The TPBLA assay

MCD_{GROWTH} (maximal cell dilution allowing growth) assays were performed in sterile 48-well LB agar plates (Greiner Bio-One, cat. 677102) prepared prior to the assay. Tetracycline (10 µg mL^{-1} final concentration) and filter-sterilised L-arabinose (final concentration of 0.075 % (*w/v*)) were added to 100 mL of sterile 1.5 % (*w/v*) LB agar cooled to < 50 °C. This solution (300 µL) was added into each of the first six wells (first row) of the 48-well plates. Ampicillin (10 mg mL^{-1} stock) was then added to the LB agar stock to give the required concentration for the next row of wells. This procedure was repeated until the plate contained 8 rows of LB agar containing increasing concentrations of ampicillin. β-lactamase-test protein constructs were screened over an ampicillin concentration range of either 0–140 µg mL^{-1} (20 µg mL^{-1} increments, for the three series (D53X-, D76X- and D98X-β_2m) and rabbit β_2m) or 0–280 µg mL^{-1} (40 µg mL^{-1} increments, for D76N+X variants, where X can be 1 to 5 mutations). Note that the A.U.C. value depends on the range of ampicillin concentration used and hence A.U.C. values obtained in experiments using different antibiotic range cannot be directly compared. Agar plates were left to set in a sterile environment.

A strip of colonies of fresh *E. coli* SCS1 cells (Stratagene) transformed with the appropriate plasmid was used to inoculate 100 mL sterile LB containing 10 µg mL^{-1} tetracycline. Cultures were incubated overnight at 37°C with shaking (200 rotation per min (rpm)). One millilitre of overnight culture was used to inoculate 100 mL sterile LB containing 10 µg mL^{-1} tetracycline and grown at 37°C with shaking (200 rpm) until an OD_{600} of 0.6 was reached. Expression of the β-lactamase fusion construct was induced by the addition of filter-sterilised arabinose at a final concentration of 0.075 % (*w/v*). Cultures were incubated for a further 1 h then serially diluted 10-fold into sterile 170 mM NaCl solution. Three microlitres of each dilution was then spotted onto the pre-prepared 48-well agar plates. The plates were incubated at 37°C for 18 h and the MCD_{GROWTH} was determined for each ampicillin concentration by visual inspection. The area under the MCD_{GROWTH} curve was then integrated to obtained a single value, the *in vivo* growth score (A.U.C.). This value was used to easily compare different variants in the TPBLA. At least 3 biological replicates were performed. The r values were calculated using Prism 8 (version 8.2.1), using the rank-based Spearman correlations.

Molecular biology

All the single point mutants for the D53X-, D76X- and D98X-β_2m series and the D76N-X-β_2m variants (for the TPBLA assay or for protein expression and purification) was cloned using the Q5® Site-Directed Mutagenesis Kit (New England BioLab). Rabbit-β_2m was ordered from Twist Bioscience and cloned into the pINK plasmid using NdeI and HindIII restriction (high fidelity) sites (New England BioLab).

Protein expression and purification

All proteins (D76X-β_2m series, D76N-X-β_2m variants and rabbit-β_2m) were expressed in *E. coli* as described previously [1]. Two purification protocols were used to purify the different β_2m variants. The initial protocol was performed as described in [1] and was used to purify most of the D76X-β_2m series, all the D76N-X-β_2m variants and rabbit-β_2m. Four D76X-β_2m variants could not be purified using this protocol since they had a very low refolding yield. For these variants the protocol was optimised. Anion exchange chromatography was performed in 25 mM Tris-HCl, pH 8.0, 8 M urea with a NaCl gradient (0 to 0.5 M over five column volumes), using a column packed with four pre-packed HiTrap Q HP columns (GE Healthcare). Dithiothreitol (DTT) (10 mM) was added to samples for 20 min (at ~20 °C) prior to loading onto the anion exchange column. The appropriate fractions from anion exchange were pooled and refolded by rapidly diluting (10-fold) the protein into refolding buffer (0.7 M arginine, pH 8.0). The refolded protein was dialysed four times with 25 mM ammonium bicarbonate pH 8.0, at 4°C (SnakeSkin™ Dialysis Tubing, 3.5k MWCO and 22 mm). Finally, the protein was lyophilised before being resuspended in 20 mM ammonium bicarbonate pH 8.0 and purified to homogeneity using gel filtration, as described in [1]. All proteins were judged to be more than 99 % pure using SDS-PAGE, monomeric using size exclusion chromatography (HiLoad 16/600 Superdex 75 PG), folded into a native structure at 20 ºC measured using far-UV CD (Chirascan Plus) and of the expected mass (confirming formation of the disulphide bond) using ESI-MS (Xevo G2-XS Q-TOF, Waters UK, Manchester).

***In vitro* fibrillation assays**

For fibrillation assays, protein was either stored as a lyophilised powder or as a concentrated solution at -80 °C. Lyophilised protein was dissolved in 25 mM sodium phosphate buffer, pH 7.4 to a protein concentration of *ca*. 500-600 μM and diluted to the desired concentration in the appropriate buffer. Stock solutions of protein were centrifuged at 14,000 *g* for 10 min before dilution into the appropriate buffer (25 mM sodium phosphate buffer, pH 6.2). Sodium chloride was added from a 2 M stock to make the desired ionic strength of 200 mM and 10 μM ThT and 0.01 % (*w/v*) NaN_3 were added to all experiments. Each condition was repeated 10 times in parallel from the same stock solution of protein and at least two biological repeats were performed. The assays contained 100 μl of 40 μM protein per well using Corning 96 well polystyrene microtiter plates with transparent bottom and low binding. Plates were sealed with clear polyolefin sealing film (STAR-LAB) and incubated at 37 °C for 100 h with alternated shaking at 600 rpm (350 s off shaking and 300 s with shaking for 367 cycles). ThT fluorescence was monitored at a single wavelength (excitation 440 nm and emission 480 nm) using a Fluostar Optima, BMG Labtech plate reader. T_{half} values were calculated by fitting the normalised data for each replicate to a generalised logistic function (Equation 1) and calculating the time at the midpoint of the curve. This analysis was carried out using Prism 8 (version 8.2.1).

$$Y(t) = A + \frac{K - A}{\left(1 + Qe^{-B(t-M)\frac{1}{v}}\right)} \qquad \textbf{Equation 1}$$

where A is the pre-transition baseline (lower asymptote), K is the post-transition baseline (upper asymptote), B is the growth rate and M is the time of maximal growth. Q and v are parameters which affect the transitions from and to the growth phase. Y is the normalised signal and t is time.

At the end of each assay the percent aggregated protein was determined by centrifugation on a bench top microfuge at 13,600 rpm for 10 min at room temperature, and analysis of the total protein, pellet and supernatant was determined using SDS-PAGE. The percent aggregate was then calculated by densitometry of the resulting bands using ImageJ.

Negative stain electron microscopy (EM)

The ThT aggregation assay end point samples were centrifuged at 14,000 g for 10 min and the pellet resuspended in a similar volume of HCl (one drop of 37 % HCl in 10 mL of ultra-pure water). 10 µl of this solution was placed on carbon coated copper EM grids (homemade) previously glow discharged for 30 sec. The grids were then blotted with filter paper to remove excess solvent and sample. Grids were placed onto drops of 1 % (*w/v*) uranyl acetate for 30 sec to stain. Grids were blotted again, washed with water and air-dried before analysis. The images were collected using Jeol 1400 (120 keV Lab6 filament and Gatan US1000XP 2k x 2k CCD camera).

Thermal denaturation monitored by far-UV CD

For thermal denaturation experiments, a spectrum at 25 °C was recorded. Next, the temperature was decreased to 10 or 20 °C and then increased in 5 °C steps with a setting time of 90 seconds at each temperature, up to 90 °C. At the end of the temperature ramp an additional spectrum was recorded 10 or 20 °C. Each spectrum was acquired from 190 nm to 260 nm with a step size of 1 nm with 1 second per point sampling. Two repeats were acquired for each temperature. The path length was 1 mm and samples contained 20 µM protein in 25 mM sodium phosphate buffer, pH 6.2. The data were converted into mean residue molar ellipticity (MRE), plotted as a function of temperature, and fitted to a 2-state model (Equation 2) using the software package CDPal [2],

$$E = e^{\left(-\frac{\Delta H_m}{R}\left(\frac{1}{T_m}-\frac{1}{T}\right) - \frac{\Delta C_p}{R}\left(\frac{T_m}{T}-1+\ln\left(\frac{T}{T_m}\right)\right)\right)} \qquad \textbf{Equation 2}$$

where E is the MRE value, ΔH_m is the change in enthalpy at the denaturation midpoint, T is the temperature recorded during the experiments in Kelvin, T_m is the melting temperature in Kelvin, ΔC_p is the difference in heat capacity between the two states, and R is the gas constant. ΔC_p was assumed to be independent of temperature. Because the thermal denaturation process was not fully reversible, $T_{m;app}$

values are quoted.

Equilibrium unfolding experiments monitored by fluorescence

Urea denaturation experiments were used to calculated $\Delta G°_{UN}$ values for β_2m variants. 250 mL of urea stock solutions of 25 mM sodium phosphate buffer, pH 7.4 containing 0 M or 10.5 M urea were made in 250 mL volumetric flasks. The exact urea concentration was calculated using a Celti refractometer and Equation 3.

$$[Urea] = 117.66\ (\Delta N) + 29.753\ (\Delta N)^2 + 185.56\ (\Delta N)^3 \qquad \textbf{Equation 3}$$

where ΔN is the difference in refractive index between the 0 M buffer and the 10.5 M urea buffers.

Stock solutions containing 4 μM of protein were made at urea concentrations of 0, 2, 4, 6, 8, and 10 M urea. The stock solutions were combined to make 1 mL solutions for every 0.2 M urea increment up to 10 M. The solutions were incubated overnight at 25 °C before measurement of tryptophan fluorescence on a PTI Quantamaster C-61 spectrofluorimeter. Tryptophan fluorescence was excited at 280 nm and the emission monitored at 325 nm over 60 sec. The signal over 60 sec was then averaged and normalised to the signal of the 10 M sample and then corrected for the urea dependence of tryptophan fluorescence using Equation 4.

$$y = \frac{(cx + d) + e^{\frac{G - mx}{RT}}(ax + b)}{e^{\frac{G - mx}{RT}} + 1} \qquad \textbf{Equation 4}$$

where G is $\Delta G°_{UN}$ in kJ mol^{-1}, m is the m-value in kJ mol^{-1} M^{-1}, R is the gas constant (8.314 J K^{-1} mol^{-1}), T is temperature in Kelvin (298 K), (ax + b) and (cx + d) correspond to the pre and post transition baselines, respectively. Midpoint urea values were calculated by solving the equation where $\Delta G°_{UN}$ = 0 kJ mol^{-1}. Equation 4 was then used to fit the normalised and corrected data to extract values for $\Delta G°_{UN}$ and the m-value.

Prediction algorithms of stability solubility and amyloid propensity

Structurally Corrected CamSol [3] was used with the webserver (http://www-mvsoftware.ch.cam.ac.uk) to predict protein solubility, where the pH was set at 6 and the patch radius at 10 Å. The data for each amino acid were exported and plotted using Prism 8 to compare WT- and D76N-β_2m, while the average CamSol value was used to compare different variants.

Aggrescan 3D 2.0 [4, 5] was used with the online webserver (http://biocomp.chem.uw.edu.pl/A3D/),

where the dynamic mode was activated, the mutate residues was turned off and the distance of aggregation analysis was set up at 5 Å. The data for each amino acid were exported and plotted using Prism 8 to compare WT- and D76N-β_2m, while the average Aggrescan 3D 2.0 value was used to compare different variants.

For CamSol Structurally Corrected [3] and Aggrescan 3D 2.0 [4, 5], the PDB files used as input were 1LDS [6] for WT-β_2m and 4FXL [7] for D76N-β_2m where the residue M0 was removed and the residues R97, D98 and M99 were added to 1LDS [6] to have similar number of residues to compare with D76N-β_2m. The D76X variants were created using PyMol 2.1.0.

Tango [8] was used with the webserver (http://tango.crg.es) to predict the propensity to form β-aggregates, where the pH was set at 6.2, the temperature at 310.15 K, the ionic strength = 0.2 M and the protein concentration at 0.04 M. The amino acid sequence was used as an input. The data for each amino acid were exported and plotted using Prism 8 to compare WT- and D76N-β_2m, while the average Tango score was used to compare different variants.

The value of the β-strand propensity of folded protein were obtained using [9].

Crystallography of D76X-β_2m variants

Seven β_2m variants (D76E-, D76A-, D76S-, D76G-, D76Q-, D76Y- and D76K-β_2m) were crystallised by mixing 100 nL of mother liquor (15 % (*v/v*) glycerol, 0.1 M sodium acetate pH 4.5 to 5.5, 28 % to 32 % (*v/v*) PEG 4000 and 0.2 M ammonium acetate) with 200 nL of protein (500 μM in 25 mM sodium phosphate buffer, pH 6.2) at 293 K in Swissci 96-well 3-prop plates (vapour diffusion, sitting drop). Crystals were flash-frozen to 100 K in liquid nitrogen without supplementation of additional cryoprotectants. Diffraction data were collected at the European Synchrotron Radiation Facility (Grenoble, France) at MASSIF-1 beamline and at Diamond Light Source (England) I24 beamline with a PIXEL detector. Data were processed with XDS [10] and AIMLESS [11] structures solved by molecular replacement using the program PHASER [12] and WT-β_2m as search model (PDB: 2YXF [13]). The structures were refined with Refmac (5.8.0258) [14] and manual model building and structure analysis were performed with COOT [15]. PyMol (2.3.2) was used for the preparation of the figures. The coordinates were deposited as for D76E- (PDB 7NMC), D76A- (PDB 7NMR), D76S- (PDB 7NMO), D76G- (PDB 7NMT), D76Q- (PDB 7NMV), D76Y- (PDB 7NMY) and D76K-β_2m (PDB 7NN5).

Creation of the βLa-D76N-β_2m library

The Diversify PCR Random Mutagenesis Kit (Takara) was used to synthesise a D76N-β_2m megaprimer, using forward (5′-GTGGTGGTGGCTCGA) and reverse (5′-AACCGCTCCCGGATC)

primers that anneal to the Gly/Ser linker regions up- and down-stream of the D76N-β_2m sequence. The product was purified on a 1 % (*w/v*) agarose gel and the desired band was excised and purified using Qiagen Gel Extraction Kit, according to the manufacturer's instructions. To prevent expression of wild-type βLa-D76N-β_2m after ligation, a 'stop template' plasmid was created. To this end, two stop codons were inserted into β-lactamase (amino acid positions 16 & 17) in the pMB1-βLa-D76N-β_2m plasmid using the Q5 Site-Directed Mutagenesis Kit (New England BioLab). A ten-fold excess of D76N-β_2m megaprimer was added to the βLa-D76N-β_2m stop template and splicing performed using the QuikChange Lightning Site-Directed Mutagenesis Kit (Agilent). Two microlitres DpnI was then added to each reaction (1 h at 37 °C) to remove template DNA. The product was purified using Qiagen PCR Purification Kit and 2 µL was used to transform TG1 electrocompetent cells (Lucigen) by electroporation (2.5 kV field strength, 335 Ω resistance and 15 µF capacitance). Following recovery, cells were plated onto pre-prepared LB bioassay agar plates containing 10 µg mL^{-1} tetracycline and incubated overnight at 37 °C.

Single colonies were picked for sequence analysis before the remaining colonies (approximately 10^6) were removed from the bioassay plates by addition of 10 mL LB medium and scraping off. The culture was centrifuged (10 minutes, 5 000 g) before DNA extraction using the Qiagen Midiprep Kit, according to the manufacturer's instructions.

Directed evolution and selection of β_2m variants using the TPBLA

Directed evolution bioassay assay plates were prepared containing 2.5 % (*w/v*) LB, 1.5 % (*w/v*) agar, 10 µg mL^{-1} tetracycline, 0.075 % (*w/v*) arabinose and 120 or 140 µg mL^{-1} ampicillin. SCS1 supercompetent cells (Agilent) were thawed on ice for 10 minutes and 50 µL cells transferred to a 14 mL round-bottomed transformation tube. Two microlitres of the prepared library plasmid DNA (100 ng μL^{-1}) was added to the cells and incubated on ice for 30 min before heat shocking at 42 °C for 45 sec. After 5 min incubation on ice, 950 µL SOC medium was added to cells and incubated (37 °C and 200 rpm) for 1 h. Three millilitres SOC medium was then added to the cells along with 10 µg mL^{-1} tetracycline. Cells were incubated for 4 h and β-lactamase expression then induced with 0.075 % (*w/v*) arabinose. Cells were then incubated (37 °C and 200 rpm) for 1 h. The culture was spread onto the prepared assay plates and incubated overnight at 37 °C. The day after, the colonies were selected and sent for sequencing in a 96-well plate (Eurofins). 1σ and 2σ (1 or 2 standard deviation from the mean) in Fig. 4A was calculated using all the mutation frequency found in the 209 sequences.

Prediction of the protein stability and protein aggregation using Solubis

A saturated mutation scan was performed on the sequence of D76N-β_2m, resulting in 1900 mutant sequences. All sequences were analysed using Tango [8]. APRs were determined considering Tango scores above 5 for at least 5 consecutive residues. The total Tango score was summed for the entire

sequence. The difference in Tango score ($\Delta Tango_{Mutant} = Tango^{D76N}_{Mutant} - Tango^{D76N}$) was calculated for each mutation. FoldX [16] was used to calculate the effect of each mutation on the thermodynamic stability of D76N-β_2m (PDB 4FXL [7]). First the structure was repaired using the FoldX command 'RepairPDB'. All mutations were calculated using the 'BuildModel' command with the repaired structure and input and the mutants listed in the 'individual_list.txt' file format. The column 'total energy' was used from the output file 'Average_BuildModel_RepairPDB_4FXL.fxout'.

Sequence alignment

An initial selection of β_2m sequences was obtained from a protein BLAST (https://www.ncbi.nlm.nih.gov/pubmed/2231712) search with the human β_2m sequence (UniProt P61769) as query on the NCBI's non-redundant (NR) database. Sequences corresponding to PDB structures or containing "synthetic" in the description were manually removed, as well as those from non-mammalian organisms. The remaining sequences were aligned using Clustal Omega (https://pubmed.ncbi.nlm.nih.gov/21988835/) and trimmed to keep only the region corresponding to the mature human β_2m sequence. Partial sequences with missing residues in this region were further removed to provide a set of 262 representative mammalian sequences (see SI Appendix Fig. S15). The residue conservation was analysed using an in-house developed script and the corresponding image was generated using the WebLogo (https://pubmed.ncbi.nlm.nih.gov/15173120/) server implemented at https://weblogo.berkeley.edu.

SI Appendix Table S1. Experimental differences in stability and aggregation kinetics of WT- and D76N-β_2m

	**WT-β_2m**	**D76N-β_2m**
**$T_{m,app}$ (°C)**	65.2 ± 0.4	53.8 ± 0.2
**$\Delta G°_{UN}$ (kJ mol^{-1})**	-29.2 ± 0.2	-18.6 ± 0.2
**m value (kJ mol^{-1} M^{-1})**	5.05 ± 0.29	4.84 ± 0.37
**T_{half} (hours)**	-	13.7 ± 1.6

The $T_{m;app}$ was measured at pH 6.2. The $\Delta G°_{UN}$ and m value were measured at 25 ºC and pH 7.4. The T_{half} was measured at 37 ºC and pH 6.2. “-“ indicates no fibrils (no significant increase in ThT fluorescence occurred during the 100 h time course of the experiment). Error bars are the fitting errors.

SI Appendix Table S2. Solubility and aggregation propensity of WT- and D76N-β_2m predicted by different algorithms

	WT-β_2m	D76N-β_2m
**Protein solubility / Structure**		
CamSol [3] (Structure corrected score)	0.917594	0.865713
Aggrescan 3D [4, 5] (Average score)	-1.1797	-1.1099
SOLart [17, 18] (Scaled solubility value)	81.1 %	75.2 %
**Aggregation propensity / Sequence**		
Tango [8] (Total aggregation score)	784.84	784.62
SALSA [19] (Area under the curve of SALSA score)	3070.3565	3070.3565
**Aggregation propensity / Structure**		
PASTA 2.0 [20] (Best energy)	-4.74108	-4.74108
FoldX [16] (Energy difference in kcal mol^{-1})	-0.738971	1.10492
SDM [21] (predicted $\Delta\Delta G$)	-0.17	-0.22

The PDB codes used were 1LDS [6] for WT-β_2m and 4FXL [7] for D76N-β_2m, where the residue M0 was removed and the residues R97, D98 and M99 were added to 1LDS [6] to have similar number of residues to compare with D76N-β_2m. The conditions were adjusted to match those used here experimentally (40 μM protein, 20 mM sodium phosphate buffer, 115 mM NaCl, pH 6.2 and 37 °C) as far as possible.

SI Appendix Table S3. Yield, stability and aggregation rates of the twenty D76X-β_2m variants

	Yield (mg / L culture)	$T_{m;app}$ (°C)	T_{half} (hours)	% insoluble fraction
**WT-β_2m**	40.0	65.2 ± 0.4	-	0
**D76E-β_2m**	21.7	58.0 ± 0.2	65.3 ± 21.2	39.8
**D76A-β_2m**	13.0	56.6 ± 0.4	29.3 ± 13.6	57.4
**D76T-β_2m**	8.6	53.5 ± 0.8	23.1 ± 8.2	79.7
**D76S-β_2m**	7.0	54.9 ±0.2	16.1 ± 9.2	81.4
**D76V-β_2m**	2.0	51.6 ± 0.2	20.2 ± 4.4	66.1
**D76N-β_2m**	15.0	53.8 ± 0.2	9.6 ± 3.8	97.5
**D76Q-β_2m**	4.4	52.5 ± 0.7	10.5 ± 3.1	94.9
**D76G-β_2m**	9.9	53.7 ± 0.4	17.6 ± 8.2	86.3
**D76Y-β_2m**	2.0	45.8 ± 0.9	-	22.1
**D76H-β_2m**	6.0	50.1 ± 0.4	14.4 ± 0.7	81.2
**D76M-β_2m**	1.0 (2.3)	48.7 ± 0.4	10.3 ± 1.2	19.6
**D76L-β_2m**	0.4	41.1 ± 0.4	-	29.9
**D76P-β_2m**	- (3.3)	41.4 ± 0.4	60.7 ± 6.4	32.8
**D76I-β_2m**	1.7	46.2 ± 1.4	-	39.9
**D76K-β_2m**	0.4 (0.7)	51.6 ± 0.8	14.7 ± 2.0	80.1
**D76W-β_2m**	- (2.0)	40.3 ± 0.3	-	0
**D76F-β_2m**	0.8	43.4 ± 0.8	-	67.7
**D76C-β_2m**	- (13.3)	46.2 ± 0.5	-	56.8
**D76R-β_2m**	- (0.8)	37.9 ± 0.8	-	40.5

Yield (mg pure protein / L of culture): expression and purification yield of the twenty D76X-β_2m variants listed in order of decreased *in vivo* growth score. 16 variants were successfully purified using a common protocol used for wild-type β_2m [1] (see Methods), while four variants could only be purified in sufficient yield by adaptation of the method specific to each sequence (D76P-, D76W-, D76C- and D76R-β_2m (the yields given in brackets correspond to the "optimised" protocol). **$T_{m;app}$ (°C):** protein stability determined for the twenty D76X-β_2m variants using temperature ramp monitored by far-UV CD at 216 nm (Methods). The error is the fitting error. **T_{half} (hours):** half time of protein aggregation (40 μM protein, in 25 mM sodium phosphate buffer, pH 6.2, containing 115 mM NaCl, 37 ºC, shaking) determined for the twenty D76X-β_2m variants using ThT fluorescence. The data were fitted using a sigmoidal model and the error represents one standard deviation of the T_{half} values obtained from 8-10 repeats. "-" indicates that no significant increase in ThT fluorescence was observed during the 100 h time course of the experiment. **% insoluble fraction:** percentage of insoluble protein at the end of the ThT aggregation assay determined for the twenty D76X-β_2m variants using centrifugation followed by SDS-PAGE (Methods). The experimental data for each of these variants are shown in SI Appendix Fig. S6.

SI Appendix Table S4. Crystallographic data collection and refinement statistics

	**D76E-β_2m**	**D76A-β_2m**	**D76S-β_2m**	**D76G-β_2m**	**D76Q-β_2m**	**D76Y-β_2m**	**D76K-β_2m**
**Data collection**							
Beamline	I24 (Diamond)	I24 (Diamond)	MASSIF-1 (ESRF)	MASSIF-1 (ESRF)	MASSIF-1 (ESRF)	I24 (Diamond)	I24 (Diamond)
Space group	I_{121}	I_{121}	I_{121}	I_{121}	I_{121}	I_{121}	I_{121}
PDB ID	7NMC	7NMO	7NMR	7NMT	7NMV	7NMY	7NN5
Cell dimensions							
a, *b*, *c* (Å)	54.6 28.9 67.6	55.7 28.8 62.3	53.4 28.8 63.0	55.4 28.7 62.5	54.3 28.8 67.6	51.7 27.7 64.5	51.4 27.7 60.1
α, β, γ (°)	90.0 102.1 90.0	90.0 98.4 90.0	90.0 97.0 90.0	90.0 98.5 90.0	90.0 102.0 90.0	90.0 101.24 90.0	90.0 96.7 90.0
Resolution (Å)	46.60 - 1.20 (1.22 - 1.20)	44.41 - 1.20 (1.22 - 1.20)	38.20 - 1.15 (1.17 - 1.15)	44.43 - 1.20 (1.22-1.20)	46.4 - 1.51 (1.54 - 1.51)	43.98 - 1.25 (1.27 - 1.25)	41.24 - 1.24 (1.26 - 1.24)
R_{pim} (%)	0.046 (0.306)	0.014 (0.066)	0.031 (0.916)	0.037 (0.219)	0.085 (0.716)	0.032 (0.927)	0.034 (0.405)
I / σ(I)	12.5 (3.5)	32.4 (10.2)	10.7 (0.8)	10.7 (2.9)	7.9 (3.6)	14.6 (0.9)	13.2 (1.8)
Completeness (%)	96.3 (77.0)	97.7 (92.3)	98.6 (98.4)	98.4 (97.1)	95.6 (95.7)	98.0 (81.4)	97.2 (72.4)
Redundancy	5.6 (4.0)	6.3 (5.2)	2.8 (2.8)	2.6 (2.4)	2.7 (2.3)	7.0 (5.3)	3.7 (2.7)
CC(1/2)	0.994 (0.828)	0.999 (0.988)	0.998 (0.504)	0.997 (0.911)	0.982 (0.354)	0.999 (0.448)	0.999 (0.691)
**Refinement**							
Resolution (Å)	46.60 – 1.25 (1.22 - 1.20)	44.41 - 1.20 (1.22 - 1.20)	38.20 - 1.15 (1.17 - 1.15)	44.43 - 1.20 (1.22-1.20)	46.4 - 1.51 (1.54 - 1.51)	43.98 - 1.25 (1.27 - 1.25)	41.24 - 1.24 (1.26 - 1.24)
No. of reflections	175 528 (4 957)	189 821 (7 420)	95 491 (4 461)	77 573 (3491)	41 608 (1 737)	172 185 (5 236)	85 357 (2 282)
No. of unique reflections	31 346 (1 236)	30 020 (1 429)	33 548 (1 614)	30 235 (1 458)	15 590 (767)	24 565 (988)	23 336 (844)
R_{work} / R_{free} (%)	15 / 16	13 / 16	15 / 18	14 / 17	22 / 27	16 / 21	19 / 23
**No. of atoms**							
Protein	856	842	818	852	838	853	829
Non-covalent ligands	22	0	18	0	0	0	0
Water	167	153	131	162	138	115	103
**RSMD**							
Bond lengths (Å)	0.0100	0.0223	0.0168	0.0182	0.0110	0.0431	0.0334
Bond angles (°)	1.499	2.2713	1.8928	2.1387	1.7064	3.282	2.4183
B-factor (Å^2)	15.637	16.711	20.470	15.912	14.384	19.957	12.336

Values for the highest-resolution shell are shown in parentheses.

SI Appendix Table S5. Comparison of the RMSD of WT- or D76N-β_2m and the different variants

	**RMSD *c.f.* WT-β_2m (Å)**	**RMSD *c.f* D76N-β_2m (Å)**
**D76A-β_2m**	0.157	0.281
**D76E-β_2m**	0.090	0.273
**D76G-β_2m**	0.197	0.307
**D76K-β_2m**	0.588	0.349
**D76Q-β_2m**	0.117	0.281
**D76S-β_2m**	0.131	0.268
**D76Y-β_2m**	0.565	0.326

The RMSD was calculated using all heavy atoms of the structure in PyMol. All these values are lower than 0.6 Å indicating that all structures are highly similar. The only difference observed is in the EF-loop as shown in SI Appendix Fig. S7.

SI Appendix Table S6. List of the 10 unique sequences containing substitutions at residue 76 with an improved growth score in the TPBLA

	**Amino acid substitutions**
**Restoring WT-β_2m**	**N76D**
**N76D + one amino acid change**	I46V/**N76D**<br>S57G/**N76D**<br>K58E/**N76D**<br>**N76D**/Y78F<br>**N76D**/V85E
**N76D + two amino acid changes**	R3G/**N76D**/K91E
**N76D + mutation in the APR**	I35V/**W60R**/**N76D**<br>**L65S**/K75E/**N76D**
**N76S + mutation in the APR**	**W60R**/**N76S**

The mutation N76D, which restores the WT-β_2m sequence is shown in bold, amino acid substitutions in the APR are in orange and the substitution N76S is in green.

SI Appendix Table S7. List of the 46 unique D76N+X-β_2m sequences giving rise to a high aggregation resistance in the TPBLA and that do not contain substitutions at residue 76

	in vivo growth rank order	Mutation (D76N+X-β_2m)	FoldX	Tango
One mutation in the APR **Residue 60**	24	**W60G**	1.744	-184.26
	28	Q8R/**W60G**	1.276	-184.15
	41	Y10F/**W60G**	1.066	-183.59
	40	S20P/**W60G**/K94R	6.306	-185.00
	38	I35V/**W60G**/K91E	3.386	-185.39
	9	**W60R**	0.729	-127.36
	43	I1T/**W60R**	1.773	-127.33
	31	N24D/**W60R**	1.507	-144.73
	11	S57G/**W60R**	0.080	-127.16
One mutation in the APR **Residue 62**	1	**F62S**	2.838	-346.85
	30	V27A/**F62S**/Y67N	8.160	-631.19
	37	**F62S**/S88P/K94R	1.463	-347.56
	44	**F62P**	4.187	-506.74
	27	**F62L**/H84R/W95R	18.190	-13.62
One mutation in the APR **Residue 63**	36	**Y63D**	2.555	-732.63
	33	S11P/**Y63D**/K75E	4.318	-732.92
	14	**Y63H**	1.520	-470.70
	12	K48N/**Y63H**	1.669	-472.23
	16	T4A/**Y63H**/Y67H/T86A	2.120	-708.77
	29	**Y63N**	2.406	-389.48
	23	**Y63N**/L87S	5.165	-389.48
	20	Y26D/**Y63N**/S88P	**1.929**	**-407.63**
One mutation in the APR **Residue 64**	15	**L64P**	10.003	-750.18
	18	**L64P**/E69G	10.662	-726.44
	2	N24D/**L64P**/V85A	11.856	-767.43
	21	K58R/**L64P**/I92T	11.576	-750.89
	25	T7I/L23P/H51R/**L64P**/Y67H	22.455	-766.39
One mutation in the APR **Residue 65**	10	**L65S**	3.162	-253.41
	8	**L65S**/D98G	3.061	-254.97
	6	K19R/S28P/V49A/**L65S**	10.537	-270.81
	22	R3S/T4A/N17D/**L65P**/I92V	6.254	-747.10
One mutation in the APR **Residue 66**	4	F22L/K48E/**Y66N**	3.141	-265.29
Two mutations in the APR	26	**W60R**/**L65S**	3.908	-417.62
	17	S20P/**W60R**/**F62S**	7.888	-427.24
	35	F22L/**W60R**/**Y63N**	3.179	-515.77
	46	**W60R**/**Y63H**/T68A	1.637	-374.70
	42	**W60R**/**Y63N**/N83S	1.854	-506.15
	32	**F62S**/**L65S**/D96G	6.374	-727.84
	7	**Y63H**/**L64P**/I92V	12.010	-750.13
	34	T4A/**L64P**/**L65S**/F70L	13.094	-750.18
	45	K19E/**W60R**/**L65S**/S88P	2.903	-419.01
	5	E36D/**W60G**/**Y63H**/F70L	3.377	-588.91
	39	K48E/**W60R**/**Y63H**/Q89R	1.909	-401.98
	19	I35V/N42S/**Y63Q**/**L64P**/Y78H	15.910	-750.17
	3	E47G/H51Q/**L64P**/**L65S**/K94R	12.385	-750.91
	13	N24D/Y26F/E47G/**Y63H**/**L64P**/V85A/Q89L	13.713	-764.17

The 46 unique variants containing amino acid substitutions that improve their score in the TPBLA, but do not contain substitutions at residue 76. Residues in the APR (residues 60-66) are highlighted in bold. The *in vivo* growth score of these 46 unique sequences is shown in SI Appendix Fig. S10B. The predicted

stability using FoldX (in kcal mol^{-1}) [16] and average aggregation propensity predicted using Tango [8] are shown (see also SI Appendix Fig. S10A).

SI Appendix Table S8. Protein stability and aggregation for six selected/designed D76N+X-β_2m variants

	In vivo growth score (A.U.C.)	$T_{m;app}$ (°C)	T_{half} (hours)
**WT-β_2m**	1066 ± 83	65.2 ± 0.4	-
**D76N-β_2m**	630 ± 10	53.8 ± 0.2	9.6 ± 3.8
**D76N_F62P-β_2m**	980 ± 37	53.9 ± 0.2	30.9 ± 2.2
**D76N_Y63D-β_2m**	1120 ± 40	54.2 ± 0.2	18.5 ± 3.4
**D76N_L64D-β_2m**	740 ± 106	41.2 ± 0.4	14.1 ± 5.5
**D76N_L64P-β_2m**	973 ± 24	32.2 ± 0.2	ND
**D76N_L65P-β_2m**	873 ± 42	13.1 ± 0.1	ND
**D76N_L65K-β_2m**	1027 ± 101	44.3 ± 0.6	-

ND: these values could not be determined as protein could not be purified in sufficient amounts for these experiments. - : these proteins did not aggregate over the 100 h time-course in the condition tested (40 μM protein, 25 mM sodium phosphate buffer, pH 6.2, 115 mM NaCl, 37 ºC and shaking). See Fig. 5B for the TPBLA and SI Appendix Fig. S13 for the raw data for the *in vivo* growth scores and T_m/T_{half}, respectively. Note that a larger range of ampicillin concentration was used to determine the behaviour of these improved variants (0 – 280 μg mL^{-1} (see SI Appendix Methods)) and hence the A.U.C. is greater than those shown for D76N- and WT-β_2m in 1D and Fig. 3.

SI Appendix Figure S1

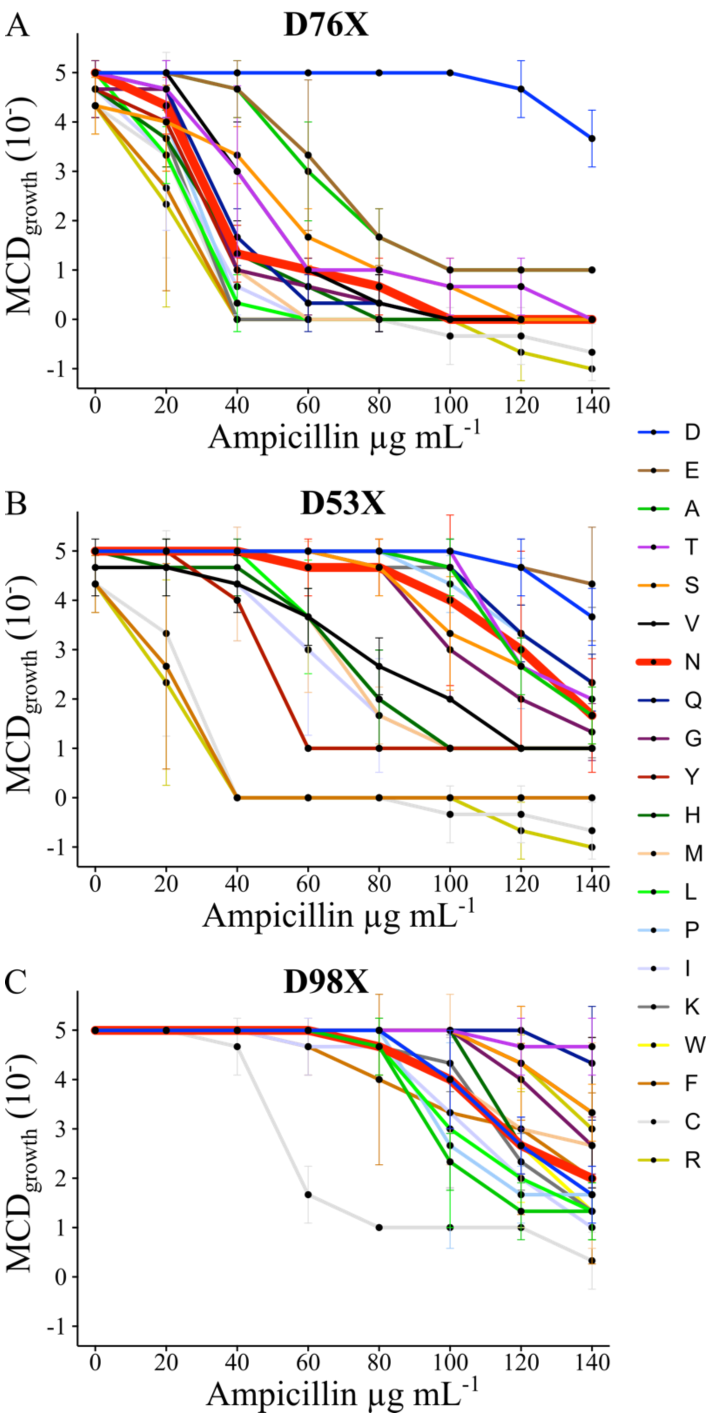

Figure S1. Antibiotic survival curves for each of the twenty variants of D53X-, D76X-, and D98X-β$_2$m. Antibiotic survival curve of the maximal cell dilution allowing growth (MCD_{growth}) on solid agar medium over a range of ampicillin concentrations for bacteria expressing the twenty variants of **(A)** D76X, **(B)** D53X- or **(C)** D98X-β$_2$m. Colour coding is indicated in the right of the figure. The error bars represent one standard deviation (n = 3 biologically independent experiments). "D" corresponds to WT-β$_2$m.

SI Appendix Figure S2.

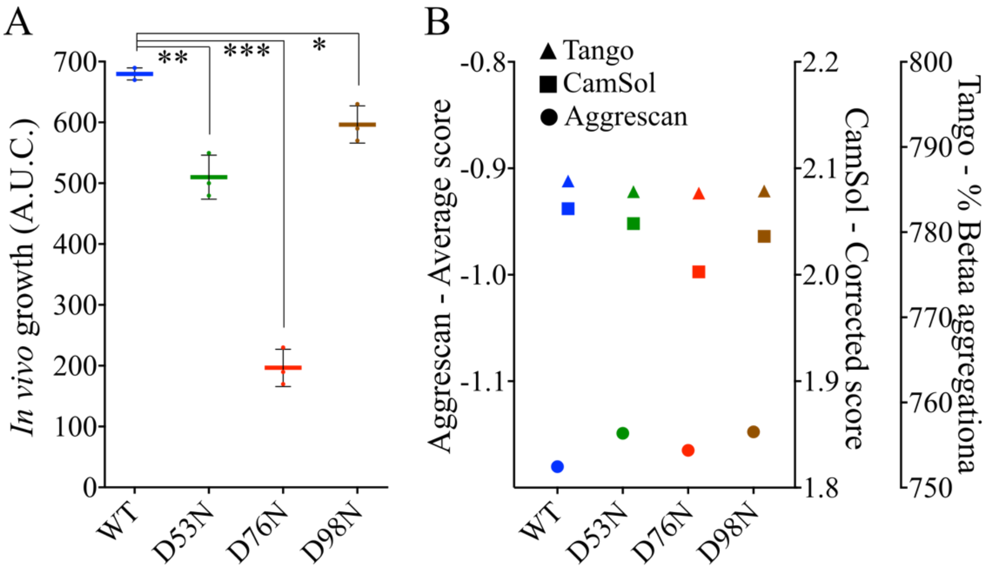

Figure S2. Comparison of the effect of Asp to Asn substitutions at residues 53, 76 or 98 in the TPBLA. (A) *In vivo* growth score (A.U.C.) of WT-, D53N-, D76N- and D98N-β$_2$m. Data represent mean values (n = 3 biologically independent experiments), where each point corresponds to one experiment. The error bars represent one standard deviation. Asterisks denote significance: * corresponds to p = 0.04, ** p = 0.01 and *** p = 0.002 (t-Test: Paired Two Sample for Means, two-tail). **(B)** Predictions of the aggregation propensity of WT-, D53N-, D76N and D98N-β$_2$m using structurally corrected Aggrescan 3D 2.0 (circles) [4, 5], structure corrected CamSol (squares) [3] and sequence-based Tango (triangles) [8]. The PDB used for these computational predictions are 1LDS [6] for WT-β$_2$m and 4FXL [7] for D76N-β$_2$m where the residue M0 was removed and the residues R97, D98 and M99 were added to 1LDS [6] to have similar number of residues to compare with D76N-β$_2$m.

SI Appendix Figure S3

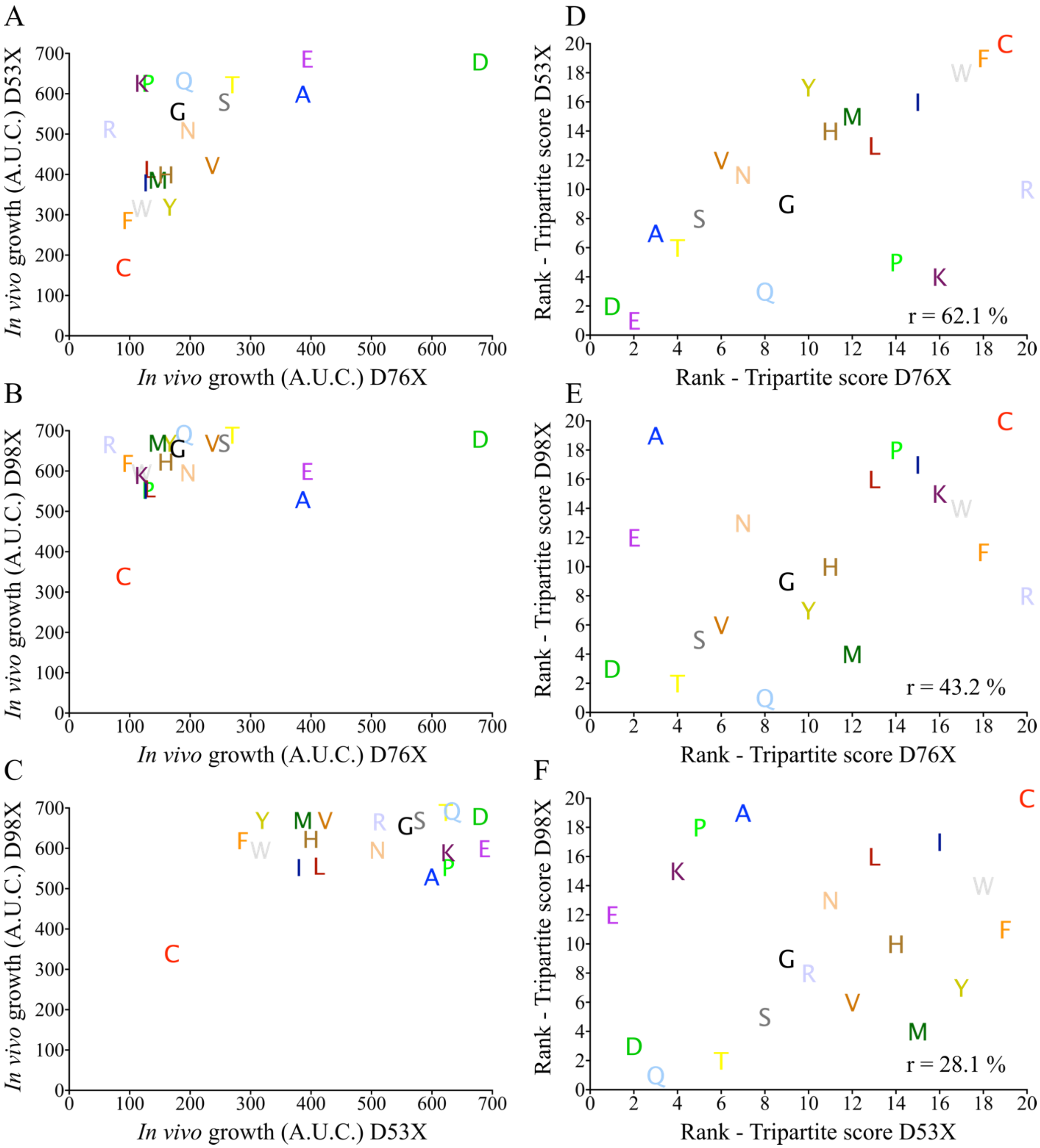

Figure S3. Correlation of the behaviour of the three β$_2$m variant series at residues 53, 76 and 98. Correlation between the *in vivo* growth scores (A.U.C) of **(A)** the D53X- and D76X-β$_2$m series, **(B)** the D98X**-** and D76X-β$_2$m series and **(C)** the D98X- and D53X-β$_2$m series. **(D-F)** As **(A-C),** but for the ranked values. The r values were calculated using the rank-based Spearman correlations for **(D-F)**. Each amino acid type is coloured the same in the six plots. "D" corresponds to WT-β$_2$m.

SI Appendix Figure S4

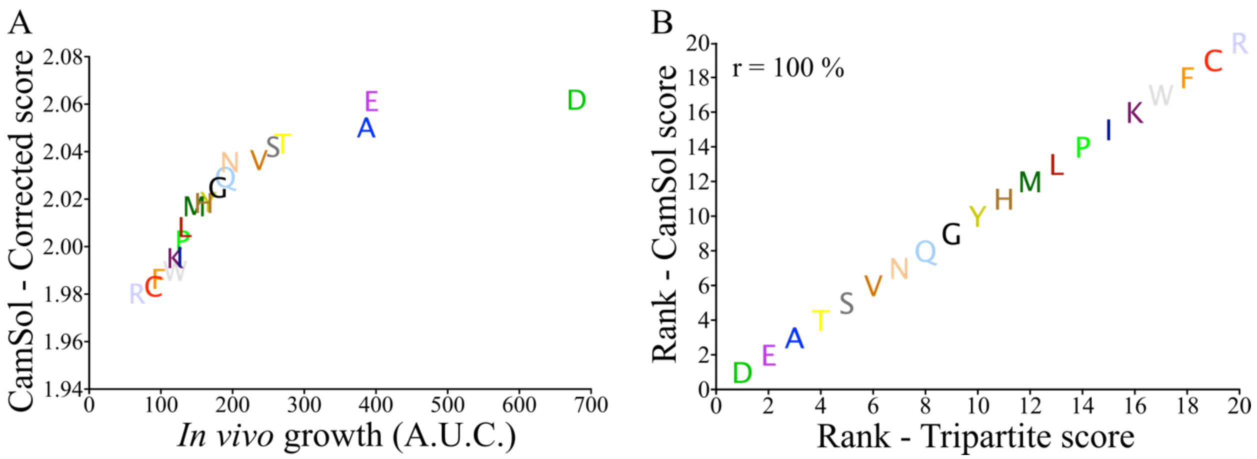

Figure S4. Correlation between the TPBLA *in vivo* growth score and the solubility predicted by CamSol for the 20 substitutions at residue 76. Correlation between **(A)** the CamSol (structure corrected) score [3] for the twenty D76X-β_2m variants and their *in vivo* growth score (A.U.C.) or **(B)** the rank order of the variants and the structure corrected CamSol score (where the highest A.U.C. score (best behaving variant) corresponds to 1 and the lowest A.U.C. score (worst behaving) corresponds to 20). Note that CamSol is an excellent predictor of the rank order of protein behaviour in the TPBLA despite the small effects of single amino acid substitutions on the average CamSol score, as shown hitherto [3]. The r value was calculated using a rank-based Spearman correlation for **(B)**. Each amino acid type is coloured the same in the two plots. "D" corresponds to WT-β_2m.

SI Appendix Figure S5

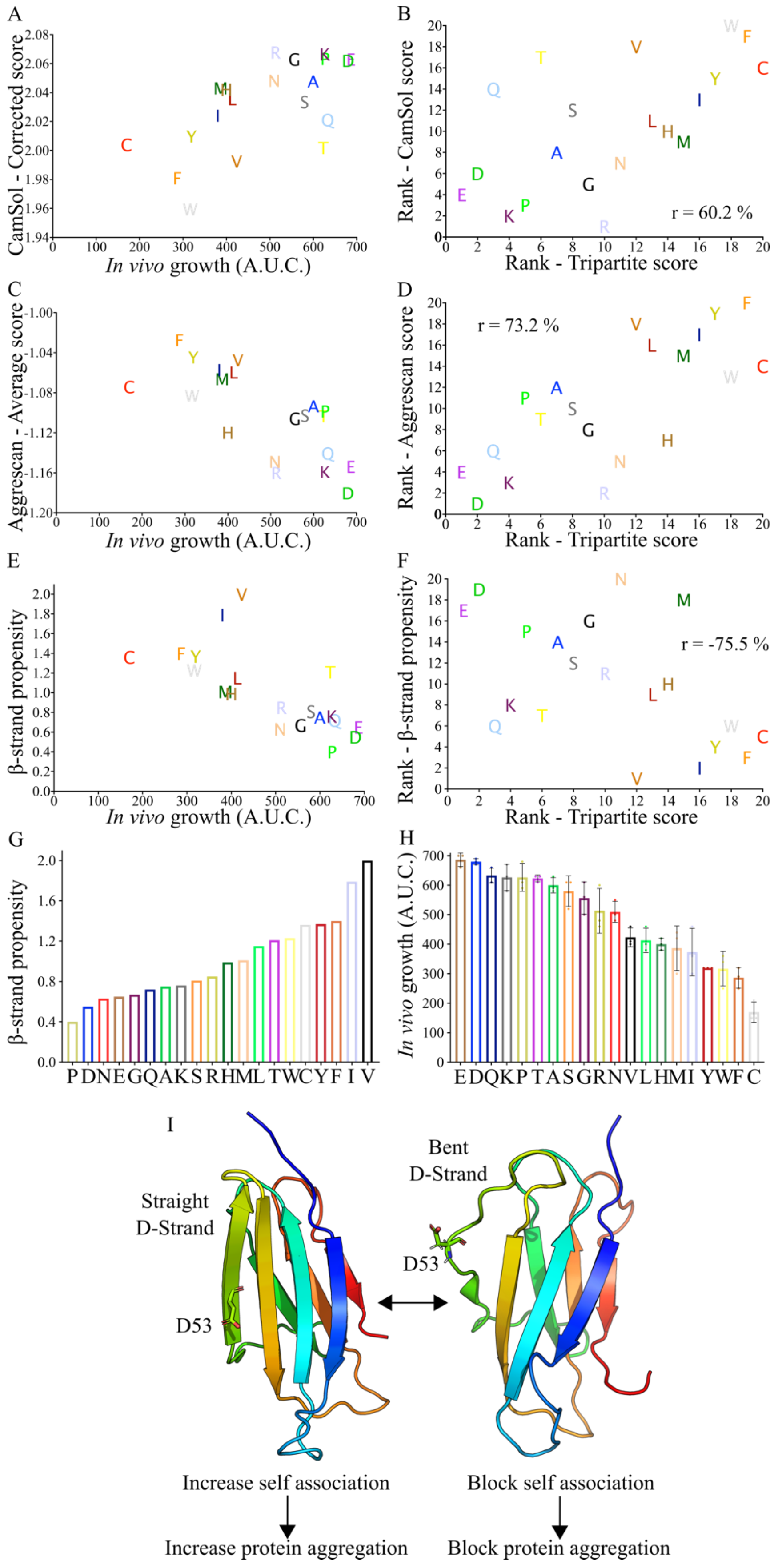

Figure S5. Correlation between the TPBLA *in* vivo growth score, and prediction of aggregation, solubility or β-strand propensity for residue substitutions at position 53. (A,B) Correlation between structure corrected CamSol [3] and the *in vivo* growth score for the D53X-β_2m series. **(C,D)** Correlation between Aggrescan 3D [4, 5] 2.0 and the *in vivo* growth score for the D53X-β_2m series. **(E,F)** Correlation between β-strand propensity [9] and *in vivo* growth score for the D53X-β_2m series. Correlation between the rank order of Aggrescan 3D [4, 5] 2.0 **(B),** structure corrected CamSol [3] **(D)** and β-strand propensity [9] **(F)** (where the lowest β-strand propensity corresponds to 1 and the highest to 20) and the rank *in vivo* growth score (where the highest A.U.C. score (best behaving variant) corresponds to 1 and the lowest A.U.C. score (worst behaving) corresponds to 20). The r value was calculated using the rank-based Spearman correlations for **(B,D,F)**. **(G)** Bar chart showing the β-strand propensity [9] for each D53X-β_2m variant and **(H)** the *in vivo* growth score (A.U.C.) for the D53X-β_2m series. Data represent mean values (n = 3 biologically independent experiments), where each point correspond to one experiment. The variants are ordered from the highest *in vivo* growth (A.U.C.) score (left) to the lowest score (right). The error bars (black) represent one standard deviation between replicates. Substitution by the same amino acid is coloured the same in all plots. The substitution "D" corresponds to WT-β_2m. **(I)** The crystal (PDB: 1LDS [6]) (left) and solution structures (PDB: 2XKS [22]) (right) of WT-β_2m. Note the different conformations of the D-strand in each structure, with the straight β-strand suggested previously as promoting aggregation [6].

SI Appendix Figure S6

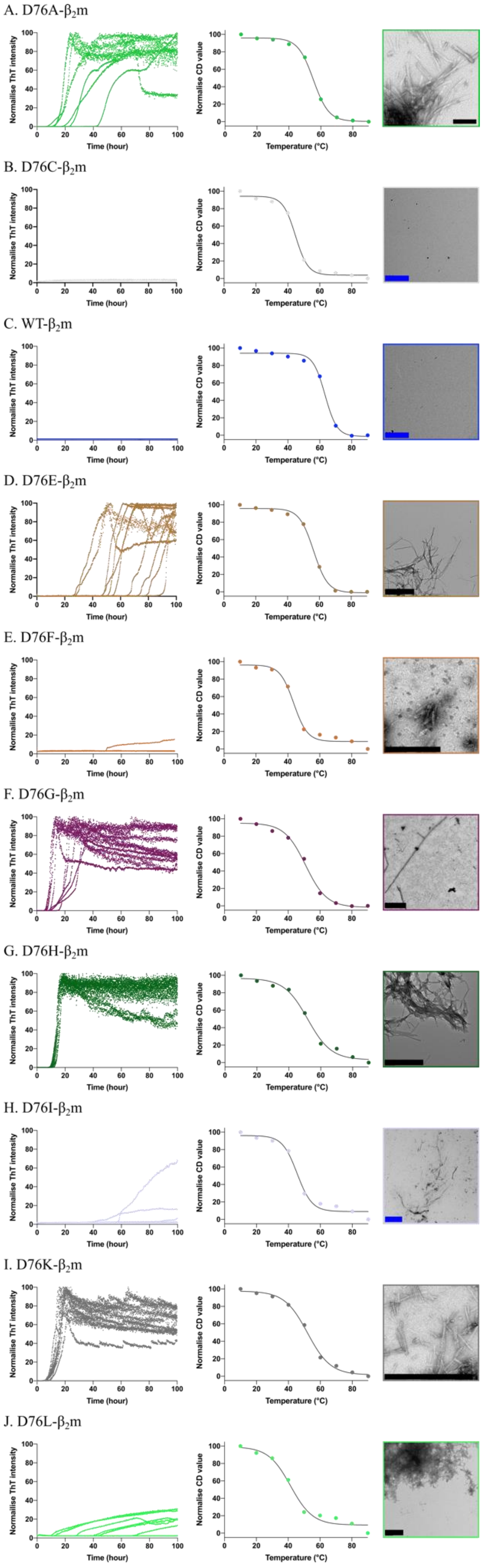

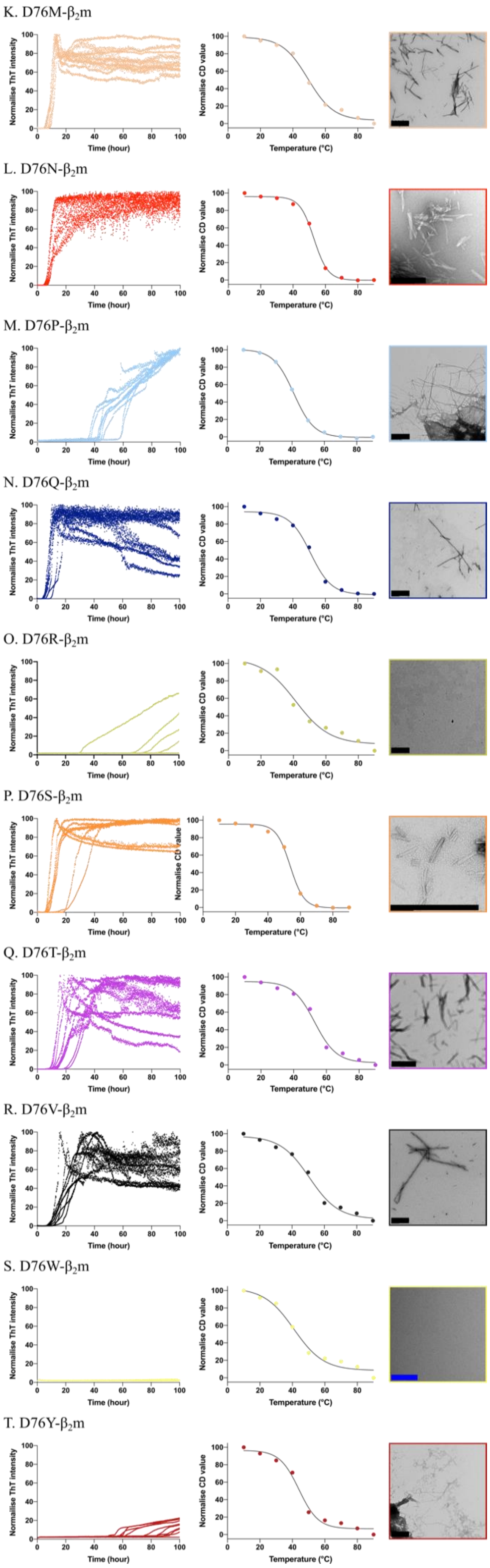
K. D76M-β2m
L. D76N-β2m
M. D76P-β2m
N. D76Q-β2m
O. D76R-β2m
P. D76S-β2m
Q. D76T-β2m
R. D76V-β2m
S. D76W-β2m
T. D76Y-β2m
Normalise ThT intensity
Time (hour)
Normalise CD value
Temperature (°C)

Figure S6. Thermal stability and aggregation rates of the twenty D76X-β_2m variants. (A-T) Aggregation kinetics monitored by ThT fluorescence (left) (between 8 to 10 replicates are shown for each variant); temperature ramp data monitored by far-UV CD at 216 nm (centre) and negative stain EM images of the twenty D76X-β_2m variants taken at the end of the reaction (100 h) (right). The scale bar in black on the EM images represents 400 nm, while that in blue represents 1 µm. T_{half} and $T_{m;app}$ values are found in SI Appendix Table S3.

SI Appendix Figure 7

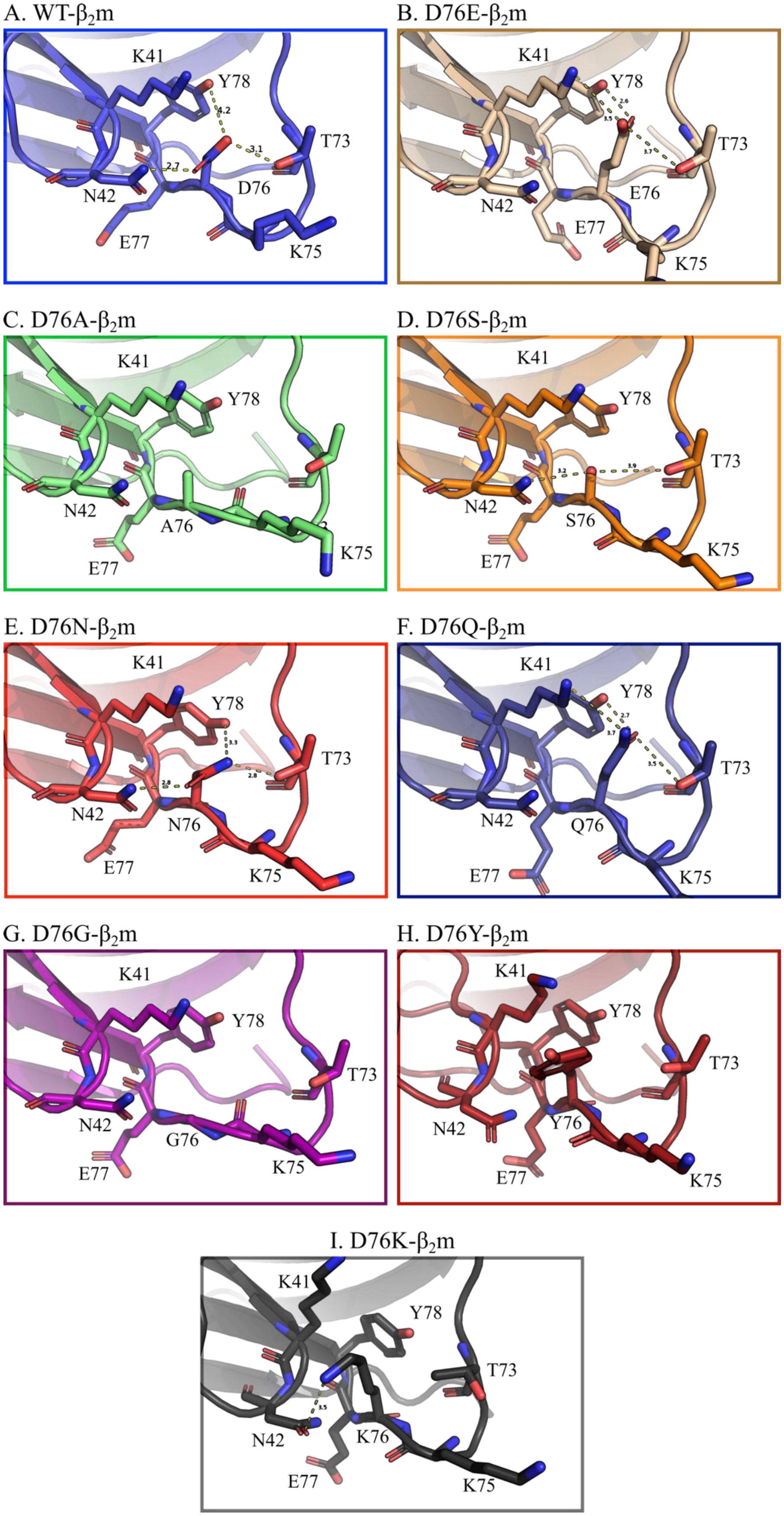

Figure S7. Hydrogen bond network at position 76 for the seven D76X-β_2m crystal structures. Cartoon representation of **(A)** WT-β_2m (PDB: 1LDS [6]); **(B)** D76E-β_2m (PDB: 7NMC); **(C)** D76A-β_2m (PDB: 7NMO); **(D)** D76S-β_2m (PDB: 7NMR); **(E)** D76N-β_2m (PDB: 4FXL [23]); **(F)** D76Q-β_2m (PDB: 7NMV); **(G)** D76G-β_2m (PDB: 7NMT); **(H)** D76Y-β_2m (PDB: 7NMY); **(I)** D76K-β_2m (PDB: 7NN5). In each case the region shown focuses on residue 76, with sidechains that form hydrogen bonds to residue 76 highlighted (N41, K42, T73, K75, E77 and Y78).

SI Appendix Figure 8

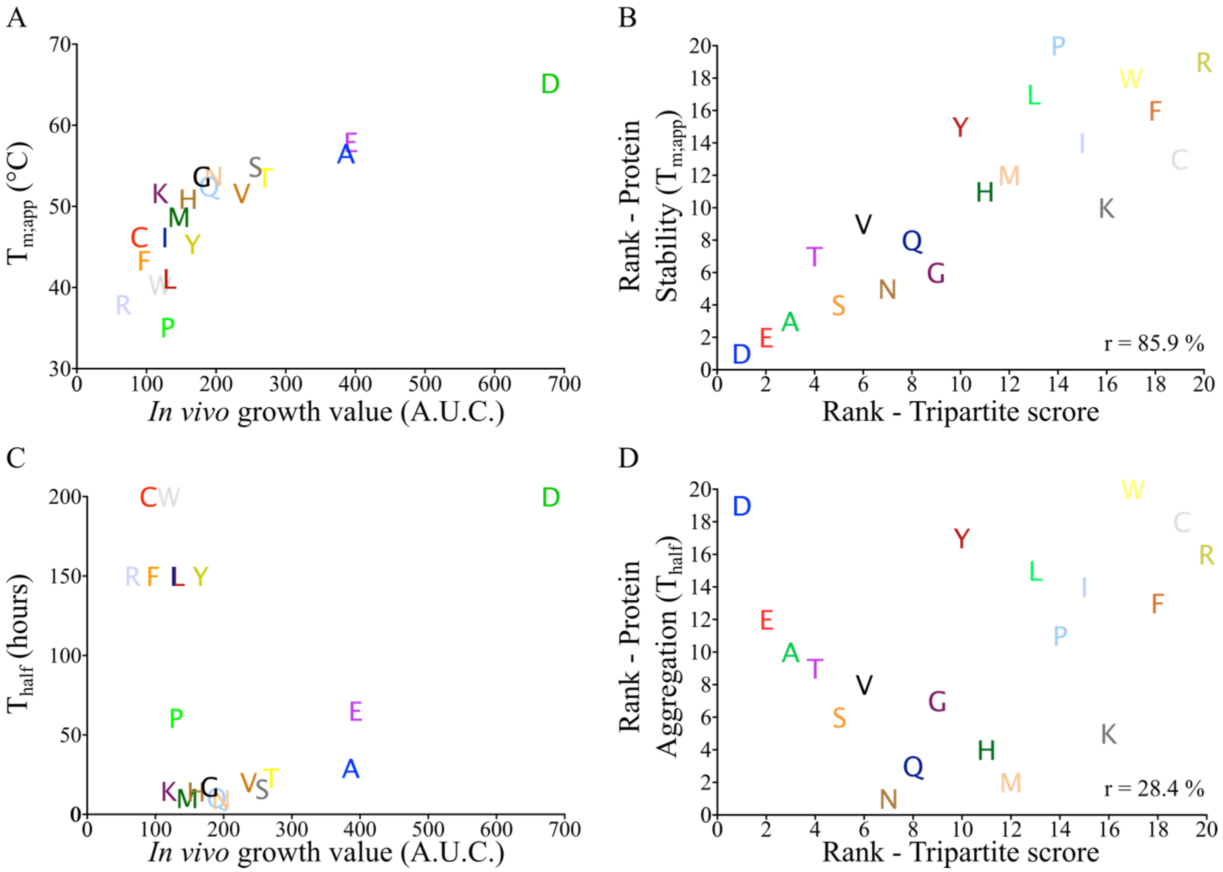

Figure S8. Correlation between thermodynamic stability/aggregation rate and *in vivo* growth score for the twenty D76X-β2m variants. Correlation between protein stability ($T_{m;app}$) **(A,B)** or protein aggregation (T_{half}) **(C,D)** and the *in vivo* growth score (A.U.C.) **(A,C)** or rank *in vivo* growth score **(B,D)** (where 1 represents the best behaving variant (high *in vivo* growth score) and 20 is the lowest score (worst behaving variant)). Each amino acid type is coloured the same in the four plots. "D" corresponds to WT-β2m. The r values were calculated using a rank-based Spearman correlation for **(B,D)**.

SI Appendix Figure S9

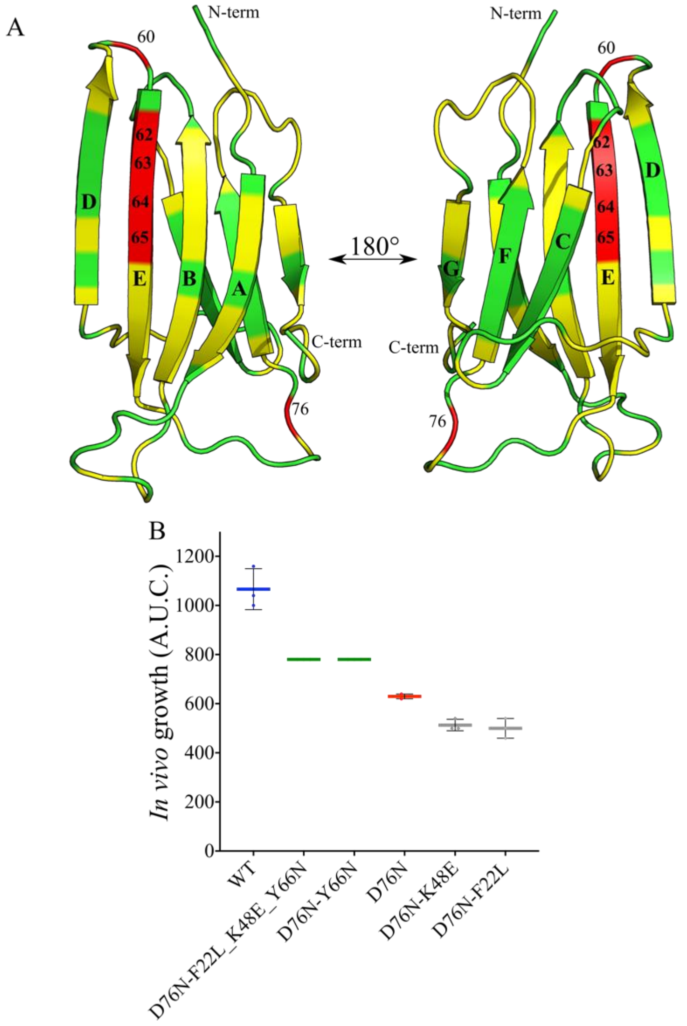

Figure S9. Residues altered in the D76N-β$_2$m* library that give rise to enhanced antibiotic resistance in the TPBLA. Crystal structure of D76N-β$_2$m (PDB: 4FXL [23]) highlighting all residues altered after random mutagenesis and selection in the TPBLA at 120 or 140 μg mL^{-1} ampicillin. Residues substituted most often are in red (> 1σ) (residues 60, 62, 63, 64, 65 and 76), residues changed less often are in yellow (< 1σ) and residues which are not altered are in green. The most frequent mutations involve residues in β-strand E. The results are colour coded according to the frequency of amino acid substitutions using 56 unique sequences (see Fig. 5A). **(B)** *In vivo* growth score (A.U.C.) of Y66N, K48E and F22L in combination with D76N-β$_2$m either together, or separately. Data represent mean values (n = 3 biologically independent experiments), where each point corresponds to one experiment. The error bars represent one standard deviation, other than for D76N-Y66N_K48E_F22L-β$_2$m for which n = 1 biological experiments. Note that a larger range of ampicillin concentration was used to determine the behaviour of these improved variants (0 – 280 μg mL^{-1} (see SI Appendix Methods)) and hence the A.U.C. is greater than those shown for D76N- and WT-β$_2$m in Fig. 1D and Fig. 3.

SI Appendix Figure S10

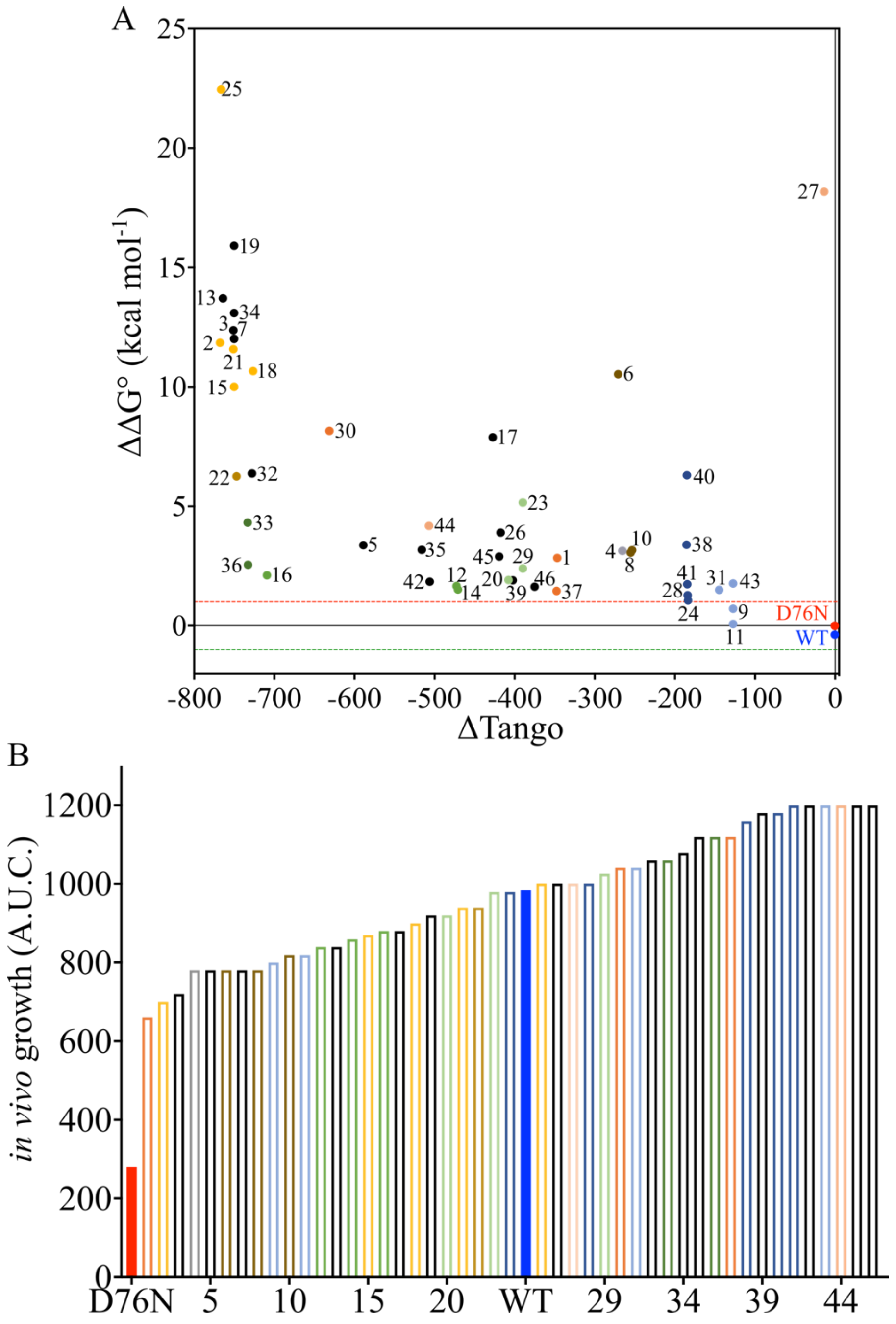

Figure S10. Unique sequences obtained during D76N-β_2m evolution and selection using the TPBLA. (A) Solubis [24, 25] was used to predict changes in protein aggregation (ΔTango) and stability (ΔΔG° kcal mol^{-1}) of these 46 variants. Each point corresponds to a single sequence (D76N + X). Green line: stabilising substitutions (ΔΔG° > -1 kcal mol^{-1}), red line: destabilising substitutions (ΔΔG° > 1 kcal mol^{-1}). **(B)** *In vivo* growth score of WT-β_2m, D76N-β_2m and the 46 unique sequences shown in SI Appendix Table S7 (i.e. excluding the 10 sequences that contain a substitution at residue 76). The clones are ranked by their *in vivo* growth score (n = 1 biological experiments). All variants have improved behaviour compared

with D76N-β_2m and 22 variants have scores that exceed that of WT-β_2m (clones 26 to 48). **(A,B)** Colour code is WT-β_2m in dark blue, D76N-β_2m is in red, D76N-F22L_K48E_Y66N-β_2m is in grey, sequences containing residue 60 mutated are in clear blue, sequences containing residue 62 mutated are in orange, sequences containing residue 63 mutated are in green, sequences containing residue 64 mutated are in yellow, sequences containing residue 65 mutated are in brown and sequences with two alterations in the APR are in black.

SI Appendix Figure S11

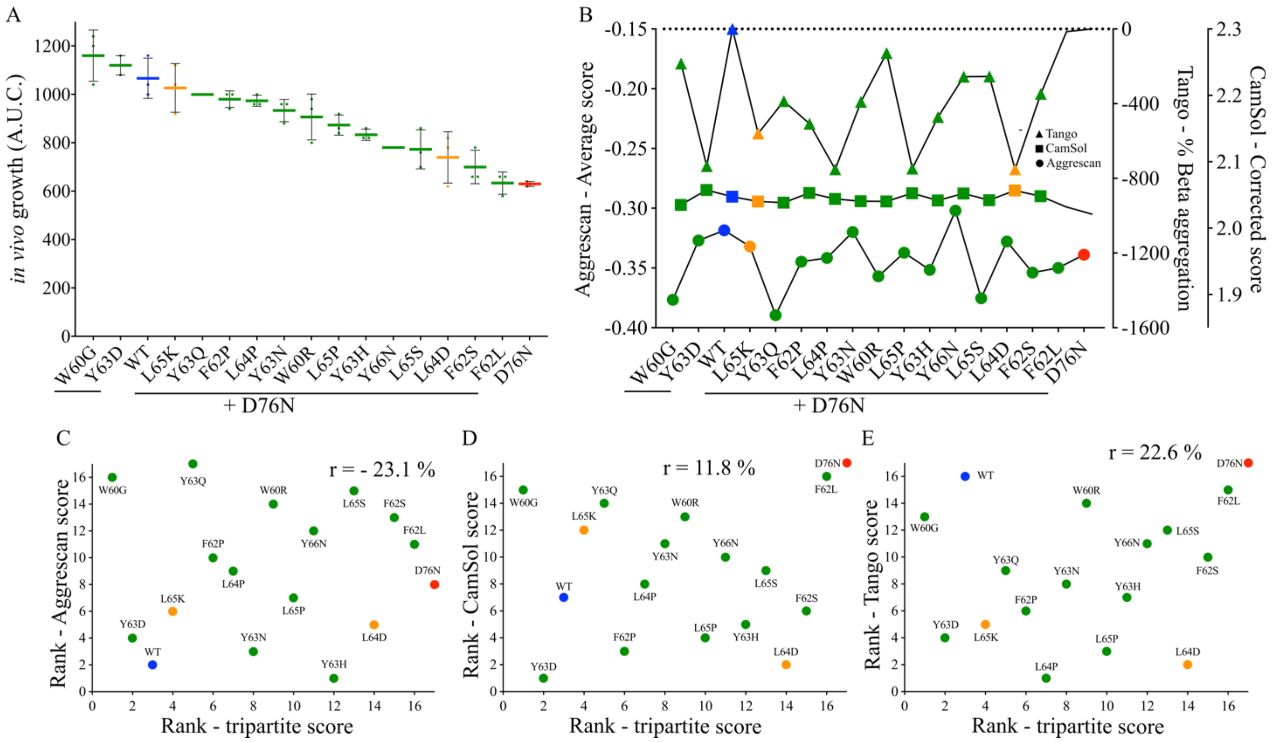

Figure S11. Prediction of the behaviour of the evolved D76N_X-β$_2$m variants. (A) *In vivo* growth scores of 15 variants (D76N + X) compared with WT- and D76N-β$_2$m. 13 variants were found in the APR (Fig. 5A, in green) and two variants were selected using Solubis (SI Appendix Fig. S12, in orange). Data represent the mean (n = three biologically independent repeats), where each point corresponds to one experiment. The variants are ordered from the highest to lowest *in vivo* growth score (A.U.C.) (left to right). The error bar represents one standard deviation. The data are reproduced from Fig. 5B for clarity. **(B)** Aggrescan 3D 2.0 [4, 5], structurally corrected CamSol [3] and sequence-based Tango [8] score for each variant shown in **(A)**. **(C,D,E)** Correlation between the rank score of Aggrescan2D 2.0 / CamSol / Tango and the rank *in vivo* growth score of the seventeen β$_2$m variants (where 1 represents the best behaving β$_2$m variants (highest *in vivo* growth score or highest stability and 17 represents the worse behaving β$_2$m variants (lowest *in vivo* growth score or lowest stability). The r values were calculated using the rank-based Spearman correlations for **(C,D,E)**.

SI Appendix Figure S12

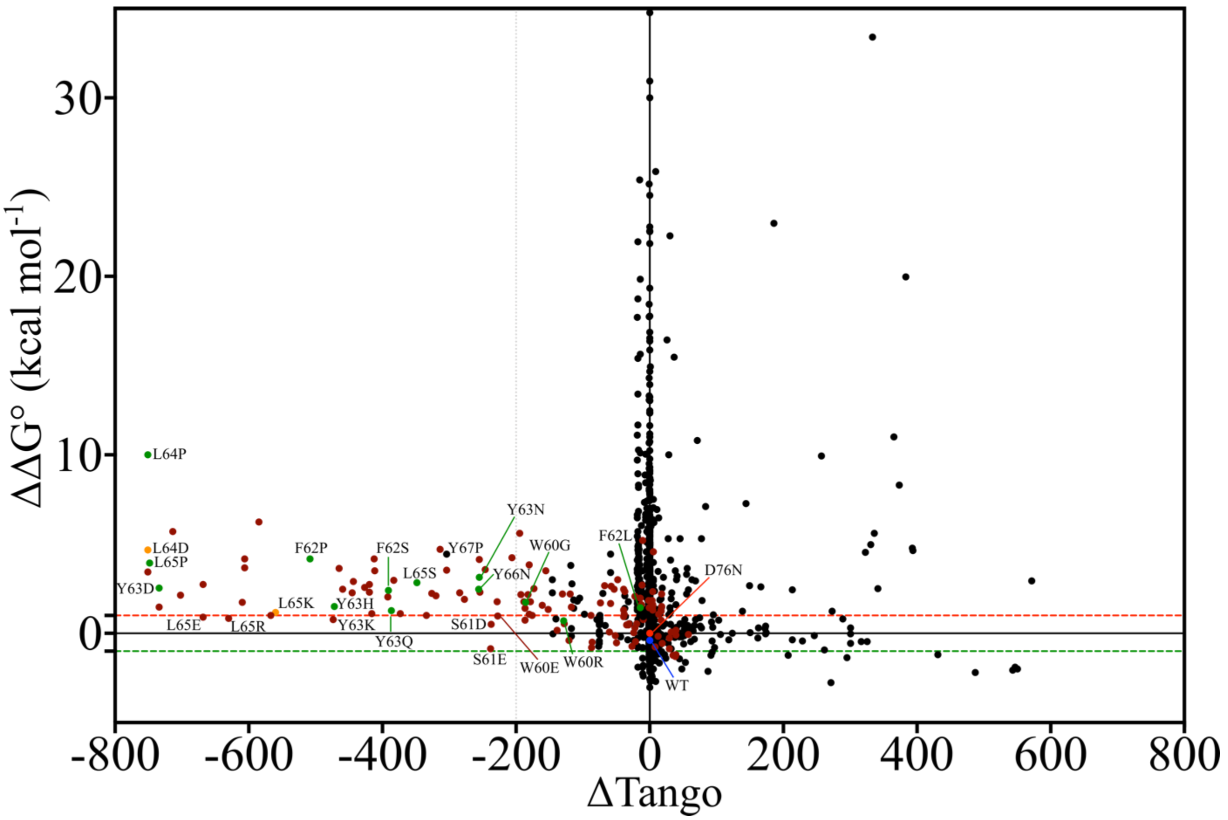

Figure S12. Saturation mutagenesis of D76N-β_2m using Solubis. Solubis [24, 25] predictions for all possible single amino acid substitutions on the aggregation propensity and stability of D76N-β_2m, where each variant was compared with D76N-β_2m. Protein aggregation is predicted using Tango [8] (where $\Delta\text{Tango}_{(X)}$ = Tango $\text{score}_{(D76N + X)}$ – Tango $\text{score}_{(D76N)}$, where X is a single residue substitution) and protein stability is predicted using FoldX [16] (where $\Delta\Delta G°_{(X)} = \Delta G°_{(D76N + X)} - \Delta G°_{(D76N)}$). Amino acid substitutions in the APR (residues 60 to 66) which were not found experimentally are in dark red, those found by random mutagenesis and selection in the TPBLA are in green and labelled, those designed using Solubis and characterised here (D76N_L64D- and D76N_L65K-β_2m) are in orange and labelled, D76N-β_2m is in red and labelled and WT-β_2m is in blue and labelled. Six single residue substitutions which reduce the aggregation propensity (ΔTango < -200) and do not significantly alter that stability of D76N-β_2m are labelled (-1< ΔΔG° < 1, L65E, L65R, Y63K, W60E, S61D and S61E).

SI Appendix Figure S13

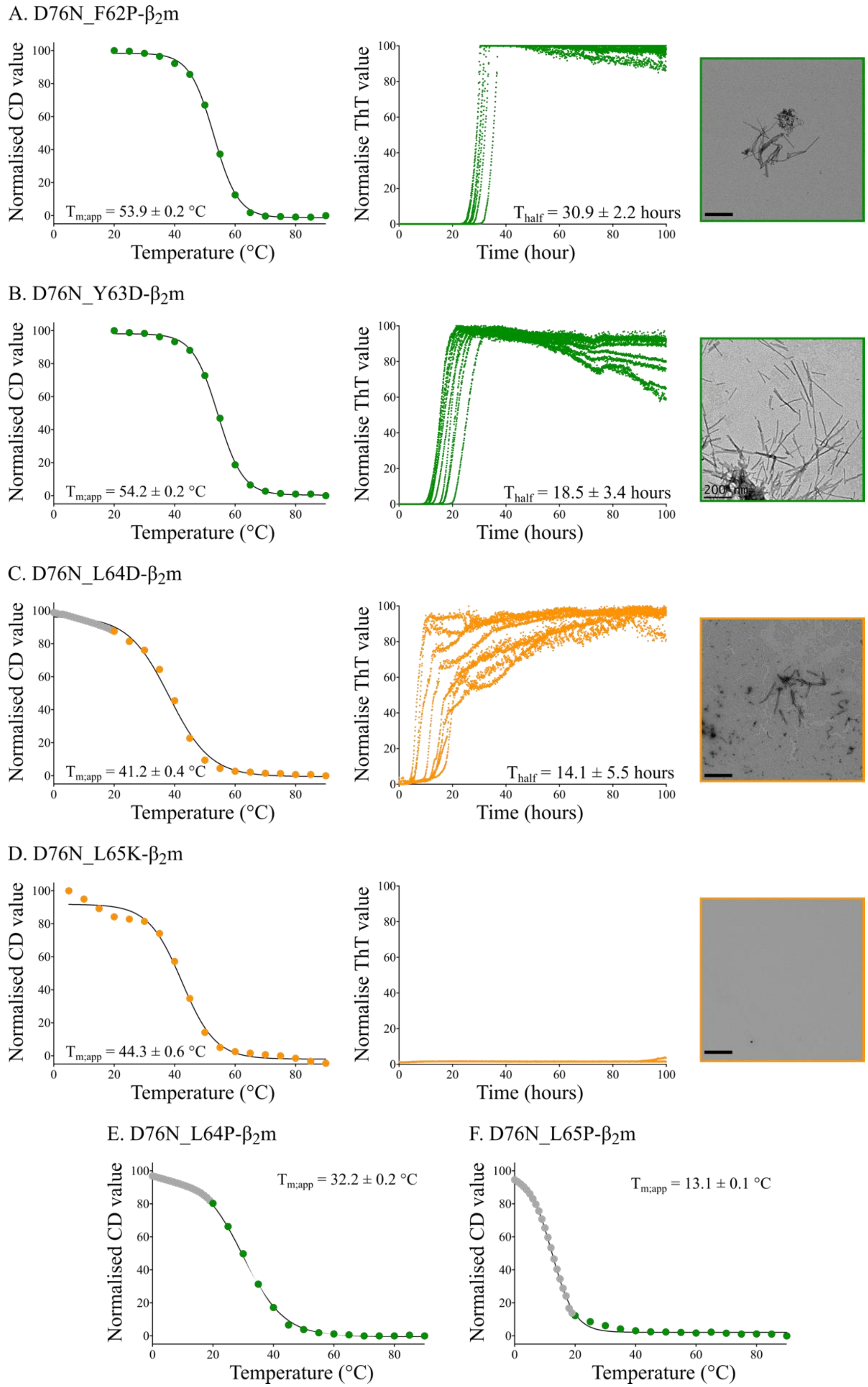

Figure S13. Characterisation of different D76N_X-β_2m variants. (A-D) Thermal denaturation monitored by far-UV CD at 216 nm (left), ThT fluorescence monitoring amyloid formation for different variants, as indicated (8-10 replicates are shown) (centre) and negative stain EM micrographs of the endpoint from each reaction (right). Scale bar = 200 nm. (**E-F)** Thermal denaturation monitored by far-UV CD at 216 nm of (**E**) D76N_L64P- and (**F**) D76N_L65P-β_2m. In **(C,E,F)**, the variants were too unstable to fit the curves independently, we used as a reference the normalised value from the pre-transition baseline of D76N-β_2m (SI Appendix Fig. S6L) (shown as grey dots). The curves were fitted using CDpal [2]. Values of the $T_{m,app}$ and T_{half} are shown in each plot.

SI Appendix S14

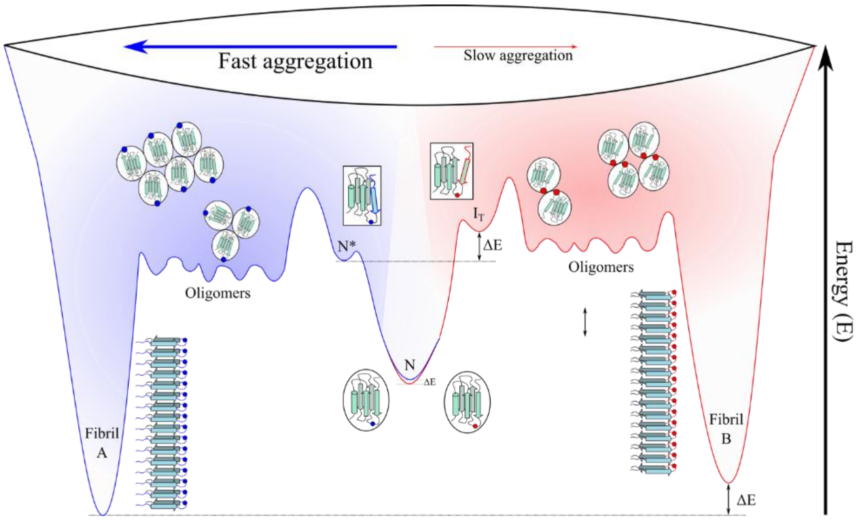

Figure S14. Schematic illustration of the energy landscape of aggregation showing how sequence changes can alter the rate and/or products of aggregation. The red and the blue dots depict different amino acid substitutions which do not affect the native structure (N), but can change the rate of aggregation, the mechanism of aggregation (different intermediates and/or oligomers are formed), and/or the products of aggregation (different fibril structures result). This complex energy landscape highlights the need to analyse all species formed on the reaction coordinate in order to understand, and predict, how changes in the sequence (resulting from familial mutation, truncation, or other post-translational modification(s)) and/or changes in the solution or cellular conditions affect amyloid formation. For WT-β_2m and D76N-β_2m, represented here by the red and blue dots, respectively, aggregation proceeds slowly/rapidly via formation of the non-native, but partially structured I_T or N* species, that are necessary precursors of their aggregation. How the mutation of Asp76 to Asn alters the mass and structure of oligomers formed and/or the structure of the fibril products remains to be resolved.

SI Appendix Figure S15

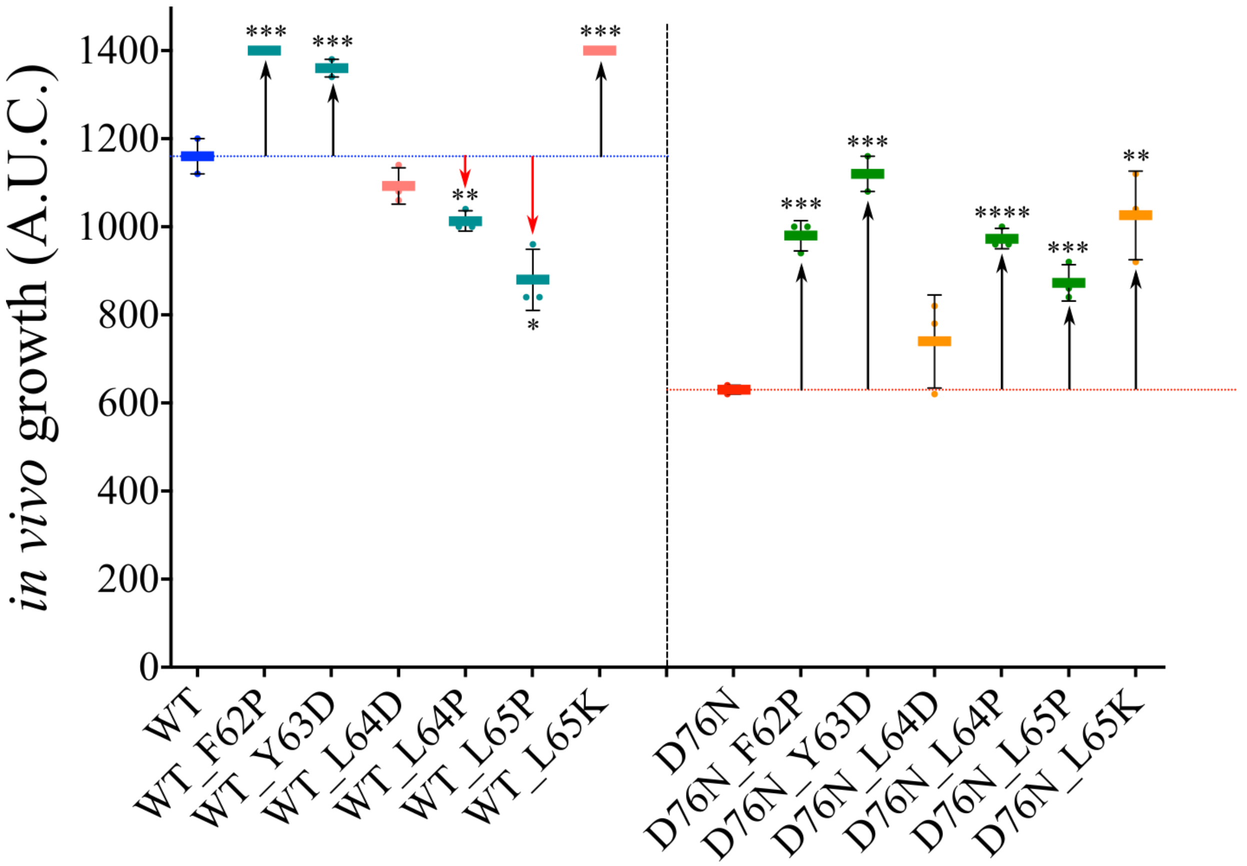

Figure S15 Comparison of the effect of single point substitutions in the APR for WT- and D76N-β_2m. *In vivo* growth score for different sequence alterations in the APR of WT-β_2m and D76N-β_2m determined using the TPBLA. The error bars represent one standard deviation (n = 3 biologically independent repeats), where each point corresponds to one experiment. The error bars represent one standard deviation. Asterisks denote significance compared with WT- (left) or D76N-β_2m (right). * corresponds to p = 0.03, ** corresponds to p = 0.02, ** 0.009 > p > 0.002 and **** p = 0.0007 (t-Test: Paired Two Sample for Means, two-tail). The blue dotted line corresponds to the mean A.U.C. of WT-β_2m. The red dotted line corresponds to the mean A.U.C. of D76N-β_2m. The black arrows show an increase of in A.U.C., while the red arrows show a decrease in A.U.C. Note that a larger range of ampicillin concentration was used to determine the behaviour of these improved variants (0 – 280 µg mL^{-1} (see SI Appendix Methods)) and hence the A.U.C is greater than those shown for D76N- and WT-β_2m in main text Fig. 1D and Fig. 3.

SI Appendix Figure S16

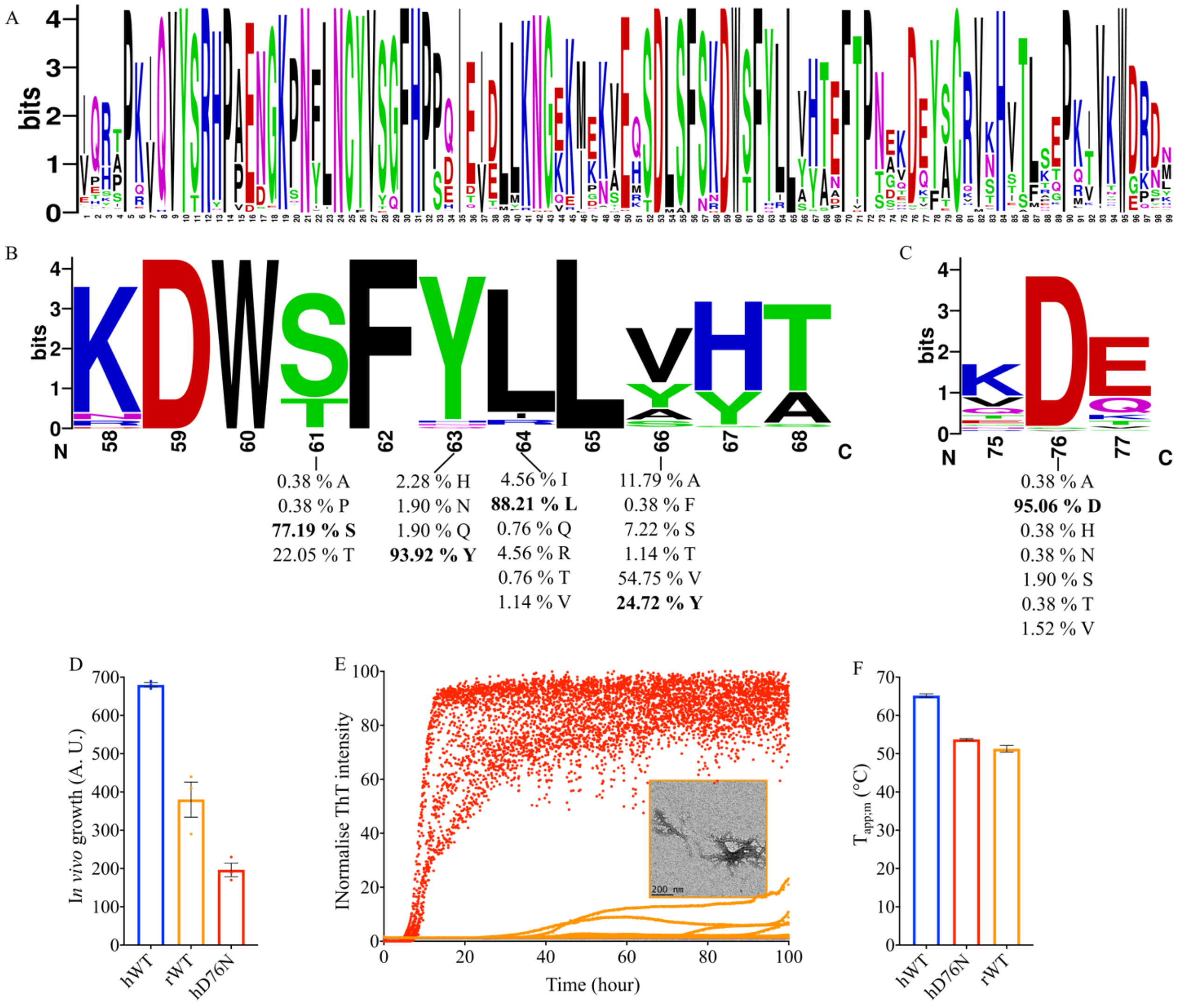

Figure S16. Natural evolution of β$_2$m sequences. (A) Consensus sequence of 262 β$_2$m sequences from Mammalia determined using WebLogo [26]. (**B**) Zoom on the sequence that contains the APR (residues 60 to 66) and (**C**) on the sequence spanning residues 75 to 77. **(D)** *In vivo* growth score of rabbit β$_2$m (rWT), compared with human WT-β$_2$m (hWT) and human D76N-β$_2$m (hD76N). The error bars (black) represent one standard deviation (n = 3 biological repeats, where each point corresponds to one experiment). **(E)** Aggregation kinetics measured using ThT fluorescence (8-10 replicates are shown). Human WT-β$_2$m (blue), rabbit β$_2$m (orange) and human D76N-β$_2$m (red). A negative stain EM image of rabbit β$_2$m taken after 100 h incubation is shown inset (fibril yield was 18.5 % compared with 97.5 % for D76N-β$_2$m) (scale bar = 200 nm). (**F**) Thermal stability of human WT-β$_2$m (hWT, blue), rabbit-β$_2$m (rWT, orange) and human D76N-β$_2$m (hD76N, red) measured using temperature ramp by far-UV CD at 216 nm. Error bars show the fitting error.

REFERENCES

1. Karamanos, T.K., et al., *Structural mapping of oligomeric intermediates in an amyloid assembly pathway*. Elife, 2019. **8**: 46574.
2. Niklasson, M., et al., *Robust and convenient analysis of protein thermal and chemical stability*. Protein Sci, 2015. **24**: 2055-2062.
3. Sormanni, P., F.A. Aprile, and M. Vendruscolo, *The CamSol method of rational design of protein mutants with enhanced solubility*. J Mol Biol, 2015. **427**: 478-490.
4. Zambrano, R., et al., *AGGRESCAN3D (A3D): server for prediction of aggregation properties of protein structures.* Nucleic Acids Res, 2015. **43**(W1): W306-313.
5. Kuriata, A., et al., *Aggrescan3D (A3D) 2.0: prediction and engineering of protein solubility*. Nucleic Acids Res, 2019. **47**(W1): W300-W307.
6. Trinh, C.H., et al., *Crystal structure of monomeric human beta-2-microglobulin reveals clues to its amyloidogenic properties.* Proc Natl Acad Sci U S A, 2002. **99**: 9771-9776.
7. Valleix, S., et al., *Hereditary systemic amyloidosis due to Asp76Asn variant beta2-microglobulin.* N Engl J Med, 2012. **366**: 2276-2283.
8. Linding, R., et al., *A comparative study of the relationship between protein structure and beta-aggregation in globular and intrinsically disordered proteins.* J Mol Biol, 2004. **342**: 345-353.
9. Fujiwara, K., H. Toda, and M. Ikeguchi, *Dependence of alpha-helical and beta-sheet amino acid propensities on the overall protein fold type.* BMC Struct Biol, 2012. **12**: 18.
10. Kabsch, W., *Xds.* Acta Crystallogr D Biol Crystallogr, 2010. **66**: 125-132.
11. Evans, P., *Scaling and assessment of data quality.* Acta Crystallogr D Biol Crystallogr, 2006. **62**: 72-82.
12. McCoy, A.J., et al., *Phaser crystallographic software.* J Appl Crystallogr, 2007. **40**: 658-674.
13. Iwata, K., et al., *High-resolution crystal structure of beta2-microglobulin formed at pH 7.0.* J Biochem, 2007. **142**: 413-419.
14. Murshudov, G.N., et al., *REFMAC5 for the refinement of macromolecular crystal structures.* Acta Crystallogr D Biol Crystallogr, 2011. **67**: 355-367.
15. Emsley, P., et al., *Features and development of Coot.* Acta Crystallogr D Biol Crystallogr, 2010. **66**: 486-501.
16. Van Durme, J., et al., *A graphical interface for the FoldX forcefield.* Bioinformatics, 2011. **27**: 1711-1712.
17. Hou, Q., et al., *SOLart: a structure-based method to predict protein solubility and aggregation.* Bioinformatics, 2020. **36**: 1445-1452.
18. Hou, Q., et al., *Computational analysis of the amino acid interactions that promote or decrease protein solubility.* Sci Rep, 2018. **8**: 14661.
19. Zibaee, S., et al., *A simple algorithm locates beta-strands in the amyloid fibril core of alpha-synuclein, Abeta, and tau using the amino acid sequence alone.* Protein Sci, 2007. **16**: 906-918.

20. Walsh, I., et al., *PASTA 2.0: an improved server for protein aggregation prediction.* Nucleic Acids Res, 2014. **42**(Web Server issue): W301-307.

21. Pandurangan, A.P., et al., *SDM: a server for predicting effects of mutations on protein stability.* Nucleic Acids Res, 2017. **45**(W1): W229-W235.

22. Eichner, T., et al., *Conformational conversion during amyloid formation at atomic resolution.* Mol Cell, 2011. **41**: 161-172.

23. de Rosa, M., et al., *Decoding the structural bases of D76N beta2-microglobulin high amyloidogenicity through crystallography and asn-scan mutagenesis.* PLoS One, 2015. **10**: e0144061.

24. Van Durme, J., et al., *Solubis: a webserver to reduce protein aggregation through mutation.* Protein Eng Des Sel, 2016. **29**: 285-289.

25. van der Kant, R., et al., *SolubiS: Optimizing Protein Solubility by Minimal Point Mutations.* Methods Mol Biol, 2019. **1873**: 317-333.

26. Crooks, G.E., et al., *WebLogo: a sequence logo generator.* Genome Res, 2004. **14**: 1188-1190.
